# The retardant effect of 2-Tridecanone, mediated by Cytochrome P450, on the Development of Cotton bollworm, *Helicoverpa armigera*

**DOI:** 10.1186/s12864-016-3277-y

**Published:** 2016-11-22

**Authors:** Lei Zhang, Yao Lu, Min Xiang, Qingli Shang, Xiwu Gao

**Affiliations:** 1 Department of Entomology, China Agricultural University, Beijing, 100193 People’s Republic of China; 2 College of Plant Science and Technology, Jilin University, Changchun, 130062 People’s Republic of China

**Keywords:** *Helicoverpa armigera*, Cytochrome P450, Plant allelochemicals, Insect development, Transcriptome

## Abstract

**Background:**

Plant allelochemicals act as toxins, inhibitors of digestion, and deterrents that affect the fecundity of insects. These compounds have attracted significant research attention in recent decades, and much is known about the effects of these xenobiotic plant secondary metabolites on insect development. To date, although ecological interactions between xenobiotic plant secondary chemicals that retard insect growth have been observed in many species, it remains unclear how particular allelochemicals influence insect development in a life stage-dependent manner.

**Results:**

We found that 2-tridecanone can affect insect development; this effect appears similar to the symptoms induced by the physiological imbalance between juvenile and molting hormones in cotton bollworm. We later detected that a decrease in the concentration of 20-hydroxyecdysone occurred alongside the observed symptoms. We next identified the transcriptome of *Helicoverpa armigera* and eightdigital gene expression libraries for shading light on how 2-tridecanone retarded the development of cotton bollworm. The expression of *CYP314A1*, *CYP315A1*, *CYP18A1*, *CYP307A1,* and *CYP306A1* (unigenes 16487, 15409, 40026, 41217, 35643, 16953, 8199, 13311, and 13036) were found to be induced by 2-tridecanone; these are known to be related to the biosynthesis or metabolism of 20-hydroxyecdysone. Expression analysis and RNA interference studies established that the retardant effect of 2-tridecanone on the development of cotton bollworm is mediated by P450 genes.

**Conclusions:**

The candidate P450 gene approach described and exploited here is useful for identifying potential causal genes for the influence of plant allelochemicals on insect development.

**Electronic supplementary material:**

The online version of this article (doi:10.1186/s12864-016-3277-y) contains supplementary material, which is available to authorized users.

## Background

Co-evolution strategies are a common phenomenon in herbivorous insect-plant interactions [1, 2]. Insects employ various strategies to increase their performance and fitness, while plants also develop efficient strategies to defend against particular insects [3]. Host plants can produce various allelochemicals to defend against the damage of herbivorous insects [4–6]. Plant allelochemicals possess beneficial or detrimental effects on the target pests; allelochemicals with negative allelopathic effects are an important part of plant defense against herbivory [7, 8]. These compounds can influence the growth, survival, and reproduction of other organisms. For example, the phenolic aldehyde gossypol can retard the developmental of the cotton bollworm, *Helicoverpa armigera* (*H. armigera*) [6]. The resistance of wild tomato (*Lycopersicon hirsutum f. glabratum*) to several arthropods has been shown to be related to the presence of high contencentrations of 2-tridecanone (2-TD) in leaves [5, 9]. 2-TD can stimulate ecdysone 20-monooxygenase activity in *Spodoptera frugiperda* [10]. 2-TD in wild tomato can defense *Manduca sexta* and also plays an important role in the plant resistance to *Leptinotarsa decemlineate*. 2-TD is also known to induce an enhanced level of tolerance to the carbamate insecticide carbaryl in *Heliothis zea* [9]. Up to now, although the phenomena of plant allelochemicals retarding the development of insects has been found in many species, details remain unclear about the life-stage dependent manner and pathway of allelochemicals to influence the insect development.

Insect pests have evolved various strategies with which to respond to allelochemicals from host plants [6]. Cytochrome P450 enzymes are a major source of adaptation to plant defense mechanisms in insects [11, 12]. Plant allelochemicals are known to induce the expression of various cytochrome P450 genes in insects. The cotton bollworm is one of the most polyphagous and cosmopolitan pest species in the world. Many studies have demonstrated that the expression levels of cotton bollworm cytochrome P450 genes can be induced by plant allelochemicals [12–14]. The expression of *CYP9A* subfamily and *CYP6AE14* genes can be induced by gossypol [12, 15]. The expression of *CYP6AE*, *CYP9A*, and *CYP6B* subfamily transcripts can be induced by xanthotoxin [16, 17]. 2-TD can significantly induce the expression of *CYP6B6* [14].

High levels of P450 gene expression are typically thought to coincide with an increased ability to metabolize exogenous compounds. Many studies have focused on detoxification enzymes that can metabolize plant natural products [18–21]. Cytochrome P450 enzymes not only act as xenobiotic detoxification agents, but also play pivotal roles in various physiological processes including the biosynthesis and metabolism of 20-hydroxyecdysone (20E) and juvenile hormone (JH), which are the major modulators of developmental processes that result in molting and metamorphosis [22].

We found that 2-TD can affect insect development, and this type of effect was similar to the symptoms induced by the physiological imbalance between juvenile and molting hormones in cotton bollworm. We later discovered that a decrease in the concentration of 20E occurred alongside the observed symptoms. We then profiled the transcriptome of *Helicoverpa armigera* and used eight digital gene expression (DGE) libraries for shading light on how 2-TD retarded the development of cotton bollworm. These results should help to deepen our understanding of how plant allelochemicals influence insect development.

## Results

### Effect of 2-TD on the development of *H. armigera*

6^th^ instar larvae were fed an artificial diet containing 2-TD (10 mg/g, W:W) to evaluate the effects of 2-TD on development. 10 mg/g 2-TD is a sublethal dosage that was selected based on our studies (Additional file 1). The pupation time of the treated group (8.4d ± 2.01) was obviously longer than that of the control (6.1d ± 1.67) (Table 1). The larval weight on the 1^st^ day of treatment with 2-TD decreased significantly compared to the control group, and the pupae weight at treatment day 10 was significantly lower than that of the control group (Table 1). The pupation rate and the adult emergence rate was significantly lower in the 2-TD treated group as compared to the untreated group (Table 1). The 20E titer in larvae was measured at 24 h. The 20E titer after treatment was suppressed to a level that was only 57% of the control level (Table 1). The adult emergence rate in the treatment group was significantly lower than that of the control (Table 1).

**Table 1:** Effect of 2-TD on larval development

Treatment group	Pupation time (d)	Pupae weight (g)	*Weight gain rate (%)	**20E titers gain rate after treated for 24 h (%)	Pupation rate	Adult emergence
Control	6.1d ± 1.67^b^	0.243 ± 0.056^a^	4.112 ± 1.578^a^	−0.75% ± 0.151 ^a^	83.33%	68.0%
2-TD	8.4d ± 2.01^a^	0.192 ± 0.049^b^	1.980 ± 1.619^b^	−32.58% ± 0.21 ^b^	45%	22.2%

*Weight gain rate, the larval weight gain rate 24 h post-treatment

**20E titer gain rate = [(the concentration of 20E after 2-TD treatment– the concentration of 20E before 2-TD treatment) / the concentration of 20E before 2-TD treatment] × 100%. Each column sharing the same superscript letter (a or b) for both treatment groups was not significantly different at *P* > 0.05

### Sequencing and sequence assembly of the *H. armigera* transcriptome

The effects of 2-TD on larval development are likely complicated and may involve several pathways and related genes. We constructed a transcriptome library of *H. armigera* with Illumina sequencing technology. The library contained 43,756,144 clean reads (101 bp + 101 bp) with an accumulated length of 4,419,370,544 nucleotides (nt) (Q20 = 98.07%), de novo assembly generated a total of 93,896 non-redundant transcripts, with a median N50 length of 597 bp**,** and finally the total number of assembled unigenes is 42,463, the median N50 length of these unigenes is 695 bp (Additional file 2). All unigenes were compared with the nonredundant (nr) NCBI protein database for functional annotation using BLASTX software with an e-value cutoff of 1e^−5^. A total of 19,382 (45.6% of all unigenes) distinct sequences matched known genes, the species distribution of unigene BLASTX matches against the nr protein database show in Additional file 3. For further quantitative assessment of the assembly and annotation completeness, we applied the software tool BUSCO (Benchmarking Universal Single-Copy Orthologs), which is based on evolutionarily informed expectations of gene content, with default settings. Out of 2675 single copy orthologs for arthropods our assembly is 27% complete (663 Complete and single-copy BUSCOs, 49 Complete and duplicated BUSCOs ), while 19% of contigs are fragmented (510 BUSCOs) and 54% are missing (1453 BUSCOs), the BUSCO analysis results show in Additional file 4. Assignments of clusters of orthologous groups (COG) were used to predict and classify the possible functions of the unigenes (Additional file 5. Among the 25 COG categories, the cluster for ‘General function prediction’ represented the largest group (1421, 19.1%) followed by ‘Translation, ribosomal structure and biogenesis’ (724, 9.76%) and ‘Replication, recombination and repair’ (683, 9.21%) (Additional file 5). Common gene ontology (GO) annotation was used to classify the putative functions of the *H. armigera* unigenes (Additional file 6). Pathway analysis of the unigenes was conducted using the Kyoto Encyclopedia of Genes and Genomes (KEGG) annotation system.

### P450 sequence alignment and phylogenetic analyses

In our study, 153 putative P450 unigene sequences were annotated by searching the nr NCBI protein database using BLASTX. 94 long P450 unigene sequences within 153 putative P450 unigene sequences and 47 full-length P450 sequences from *B. mori* were used to construct a phylogenetic tree (Additional file 7). The annotated P450 unigenes in the tree belonged to the CYP2, CYP3 (including CYP6 and CYP9), CYP4, and the mitochondrial CYP (mito.CYP) clans [23]. Among the 153 predicted P450 unigenes in *H. armigera*, 11, 11, 80, and 48 unigenes were classified into the mito.CYP, CYP2, CYP3, and CYP4 clans, respectively.

### Comparison of P450 gene expression profiles at different developmental stages

In order to determine the P450 genes involved with the effects of 2-TD on larval development, eight DGE libraries were constructed to identify unigene expression profiles for shading light on how 2-tridecanone inhibited retarded the development of cotton bollworm. After removing low-quality reads, each library generated approximately eight million clean reads. Among these clean reads, 5.8–7.3 million reads were mapped to unigenes in transcriptome libraries. The Q20 ranged from 85.57 to 97.56% (Additional file 8). qPCR was used to confirm 34 unigenes expression profile results. Compared with DGE libraries results, the accuracy of these P450 unigenes expression detected by qPCR is up to 94% (Additional file 9, Additional file 10).

**Fig. 1:**
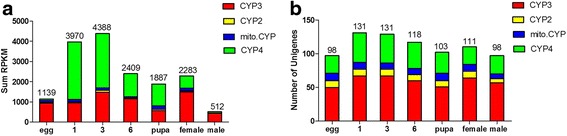
The DGE library data for the expression of P450 unigenes in different development stages. **a** Sum expression of P450s in larvae at different developmental stages. **b** The numbers of P450 unigenes at different development stages. 1: 1^st^ instar larvae; 3: 3^rd^ instar larvae; 6: 6^th^ instar larvae

**Fig. 2:**
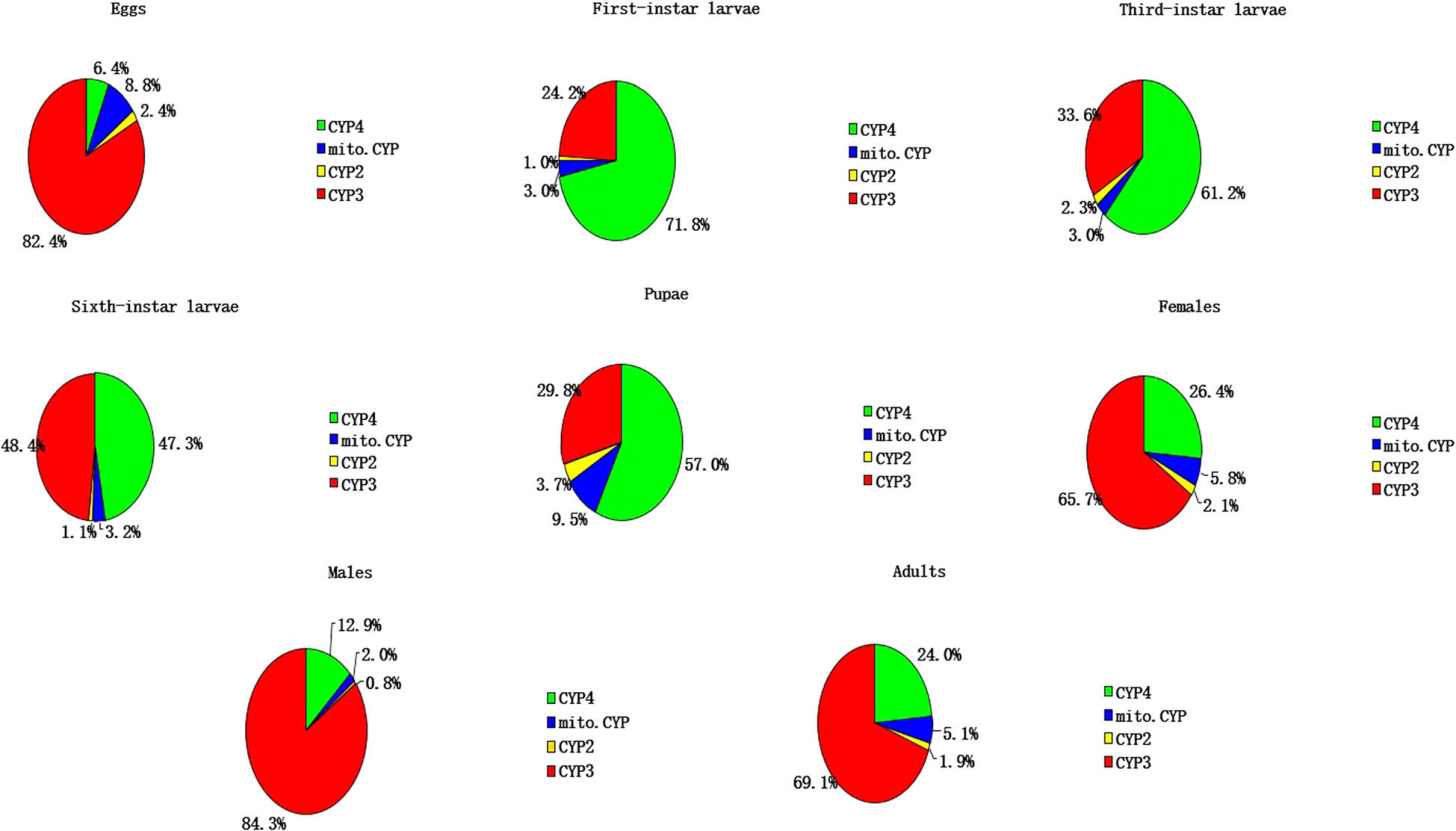
Percentage of each CYP clan expressed in different developmental stages. RPKM < 0.1 was used as the criterion to judge the unigenes were not expressed during a given developmental stage. The adults samples consisted of equal numbers of female and male individuals

Figure 1a and b show the summed expression of P450 unigenes and the numbers of P450 unigenes (including those of the CYP2, CYP3, CYP4 and moti. CYP clans) in the different development stages. The total expression amount and the numbers of P450 unigenes was higher in larvae than in the egg and adult samples, with the highest total expression level of P450s occurring in 3^rd^ instar larvae. During the transformation from eggs to larvae, the percentage of expressed CYP4 clan unigenes sharply increased from 6.4 to 71.8%, while the CYP3, CYP2, and moti. CYP clan unigenes decreased significantly from 82.4, 2.4, and 8.8% to 24.2, 1.0, and 3.0% respectively. During the transformation from larvae to pupae, the percentage of expressed annotated CYP4, CYP2 and moti. CYP clan unigenes increased dramatically from 47.3, 1.1, and 3.2% to 57, 3.7, and 9.5%, respectively; the percentage of expressed CYP3 clan unigenes decreased from 48.4 to 29.8% during this transition (Fig. 2) During the transformation from pupae to adults, the percentage of expressed annotated CYP4, CYP2 and moti. CYP clan unigenes decreased dramatically from 57, 3.7, and 9.5% to 24, 1.9, and 5.1%, respectively, while the percentage of expressed CYP3 clan unigenes increased from 29.8 to 69.1% (Fig. 2). All the expression of the 153 P450 unigenes in different DGE library were listed in Additional file 11.

**Fig. 3:**
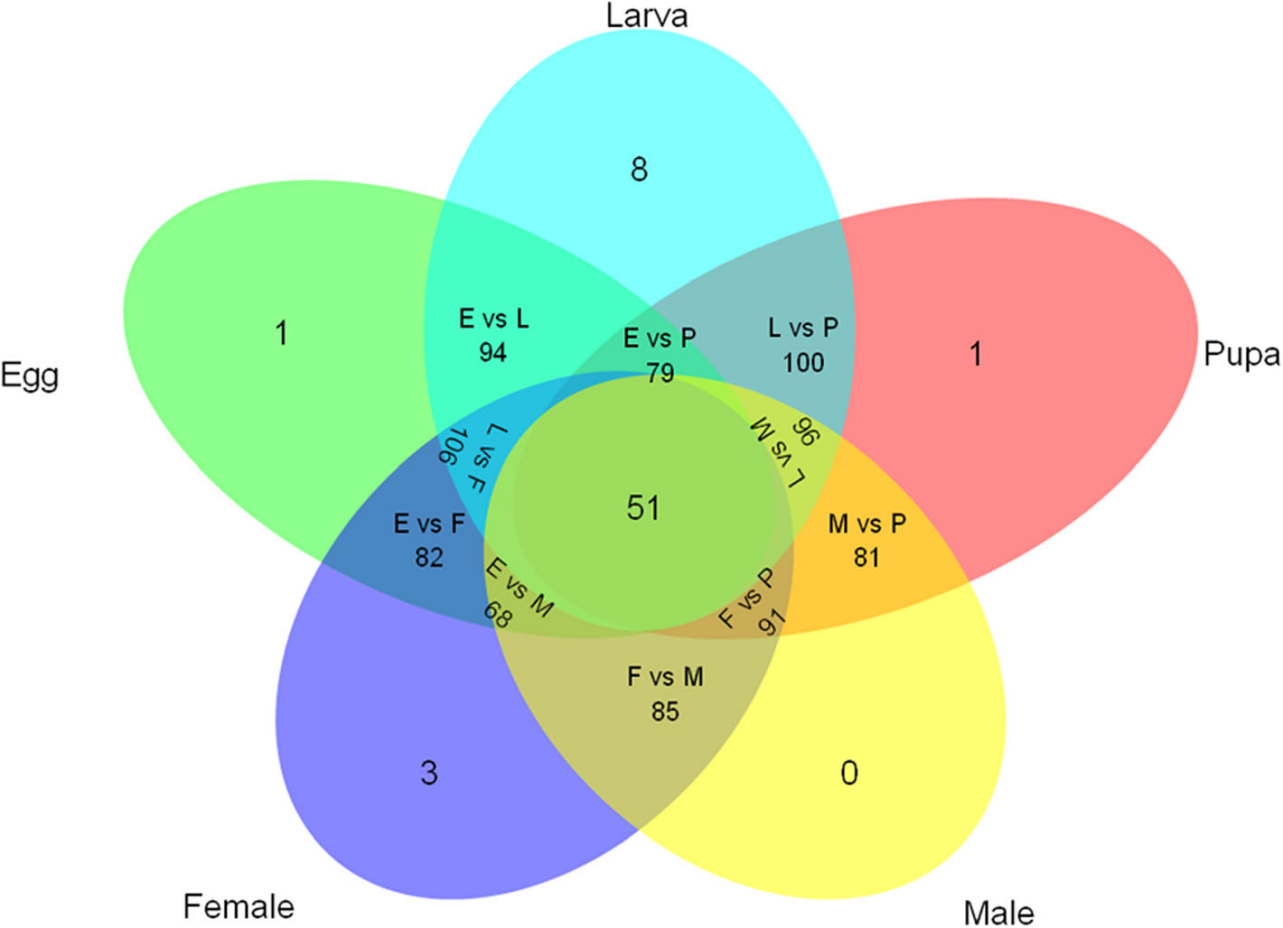
Numbers of P450 unigenes expressed in a developmental stage-specific manner. RPKM < 0.1 was used as the criterion to judge the unigenes were not expressed in a given developmental stage. E: eggs; L: larvae; P: pupae; M: males; F: females

Among the 153 annotated P450 unigene sequences, the expression of 150 unigenes (98.1%) were detected in at least one DGE library. Three P450 unigenes were not detected in any DGE library; either these were not expressed in the particular life stages that we examined or these were possibly pseudogenes. Among the expressed P450 unigenes, 33% unigenes (55 sequences) were expressed in all life stages (Fig. 3). Some P450 unigenes were specifically expressed at a particular developmental stage: eight P450 genes were specifically expressed in larvae, all of these belonged to the CYP3 or CYP4 clans. We found one specific P450 unigene that was only expressed in eggs. Likewise, a single unigene was expressed specifically in the pupae stage. Three P450 unigenes were expressed only in females. All these specifically expressed P450 unigenes belonged to the CYP3 or CYP4 clans (Table 2).

**Table 2:** P450 genes expressed at specific developmental stages

Family	Stage	Gene number	Clan	Homologous genes	% similarity, organisms	RPKM^b^
P450s^a^	Egg	32583	CYP3	6AX1.6B3	82%, *N. vitripennis*	0.72
	Larval	13679	CYP3	6B2, 6B6, 6B7	89%, *H. armigera*	0.36
		22278	CYP3	321B1	*Spodoptera litura*	1.58
		15388	CYP 3	6AE14	100%, *H. armigera*	19.16
		19180	CYP 3	9A18	74%, *H. armigera*	7.63
		12812	CYP4	4 L6	85%, *B. Mori*	1.11
		10466	CYP 4	340AA1	75%, *S. littoralis*	29.13
		10601	CYP 4	341A2	88%, *B. Mori*	3.84
		14820	CYP 4	341B1	88%, *B. mori*	3.57
	Pupa	28770	CYP 3	9A14	83%, *H. zea*	4.72
	Female	29309	CYP 3	6CV2	*Plutella xylostella*	17.73
		29201	CYP 3	6B1	*Papilio polyxenes*	16.33
		22936	CYP 4	402C1	78 %, *Bemisia tabaci*	14.07

^a^The cytochrome P450 clan schema used here follows the system proposed by Feyereisen *et al*. (2006)

^b^RPKM < 0.1 was used as the criterion to judge the unigenes were not expressed during a given developmental stage

### Effect of 2-TD on the expression of P450 genes

**Fig. 4:**
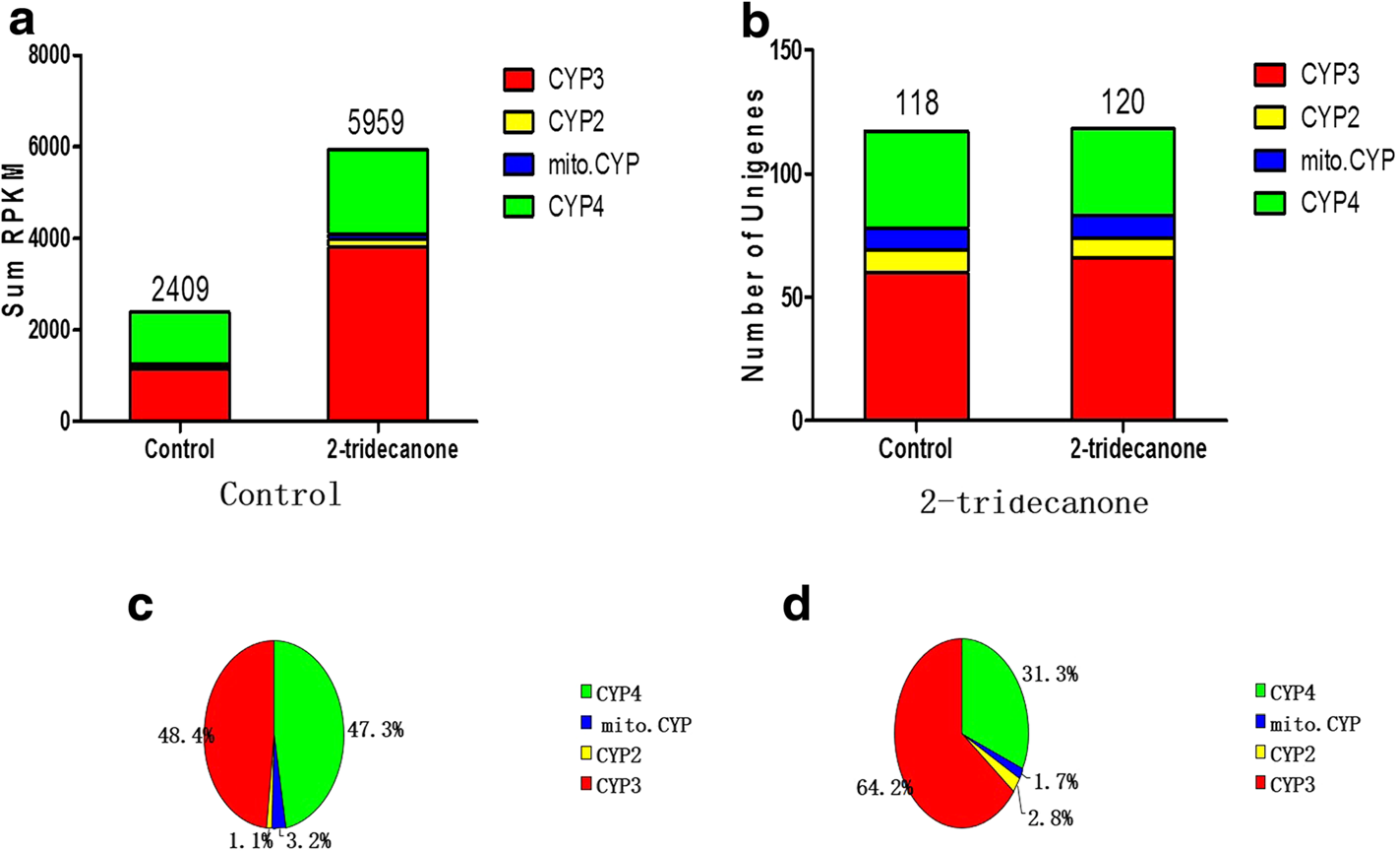
2-TD affects the expression of P450. **a** The sum of the expression of P450 unigenes in 2-TD treated and untreated groups. **b** Numbers of P450 unigenes in 2-TD treated and untreated groups. **c** The percentage of each CYP clan expressed in 6^th^ larvae. **d** The percentage of each CYP clan expressed in 6^th^ larvae treated with 2-TD. Control: 6^th^ larvae un-treated with 2-TD; 2-tridecanone: 6^th^ larvae treated with 2-TD for 24 h

Figure 4 shows the total expression levels of P450 unigenes and the numbers of P450 unigenes observed in the DGE libraries of 6^th^ instar larvae treated by 2-TD for 24 h compared with the control. The total expression levels of P450 unigenes in the larvae treated by 2-TD was 2.6 fold higher than the control group (Fig. 4a). There were more P450 unigenes expressed in the 2-TD-treated group than in the control larvae; the additional two P450 unigenes belonged to the CYP3 clan (Fig. 4b). The percentages of expressed CYP3 and CYP2 clan unigenes were obviously higher in the 2-TD-treated group (64.2 and 2.8%) than in the control (48.4 and 1.1%, respectively), while the percentages of CYP4 and mito.CYP clan unigenes were higher in the control (47.3 and 3.2%) than in the 2-TD-treated group (31.3 and 1.7%) (Fig. 4c and d).

An absolute value of Log_2_ Ratio ≥ 1 were used as thresholds to judge the differences of gene expression levels. 49 annotated P450 unigenes were up-regulated, and 22 P450 unigenes were down-regulated in larva treated by 2-TD, as compared to the control group. 7 of these annotated P450 unigenes belonged to the CYP2 clan and 7 of these P450 unigenes were classified into the mito.CYP clan; none of these 14 unigenes were uniquely expressed in a particular developmental stage. However, 15 unigenes from the CYP3 and CYP4 clans were specifically expressed in a particular stage of *H. armigera* development (Table 3).

**Table 3:** Up- and down-regulated P450 genes in cotton bollworm in response to 2-TD treatment

Family	Classification	Homologous genes	% similarity, organisms	Transcripts number	Log_2_Ratio^a^	Expression stage	2-TD induced
P450s^b^	Mitochondrial	**314A1**	**86%,** ***B. mori***	**16487**	**2.55**	**All, 3 > 1 > 6**	**Up**
		**314A1**	**83%,** ***S. littoralis***	**15409**	**−2.29**	**All, 3 > 1 > 6**	**Down**
		333B3	69%, *S. littoralis*	12317	−1.12	All, 1 > 6 > 3	Down
		333A3	75%, *S. littoralis*	25319	1.44	All, 3 > 1 > 6	Up
		**315A1**	**73%,** ***S. littoralis***	**40026**	**−1.07**	**All, 3 > 1 > 6**	**Down**
	Clan 2	**18A1**	**72%,** ***S. littoralis***	**41217**	**4.12**	**All, 6 = 3 = 1**	**Up**
		**18A1**	**81%,** ***S. littoralis***	**35643**	**5.06**	**3 > 1, pupa, female**	**Up**
		**18A1**	**76%,** ***S. littoralis***	**16953**	**4.73**	**3 > 1**	**Up**
		15C	71%, *B. mori*	14800	1.53	1, female	Up
		**306A1**	**82%,** ***S. littoralis***	**13036**	**−3.32**	**All, 3 > 1 > 6**	**Down**
		**307A1**	**99%,** ***H. armigera***	**8199**	**−4.55**	**3 > 6 > 1, pupa**	**Down**
		**307A1**	**99%,** ***H. armigera***	**13311**	**−4.92**	**Egg, 3 > 6, pupa**	**Down**
	Clan 3 (include CYP6 and CYP9)	6B2,6B6,6B7	96%, *H. armigera*	18705	3.08	Egg, female	Up
		6B2, 6B6, 6B7	94%, *H. armigera*	40306	2.23	All, 3 > 1 > 6	Up
		321A2	98%, *H. zea*	17923	7.79	No expression	Up
		6B2	96%, *H. armigera*	2844	3.03	All, 6 > 3 > 1	Up
		6B2, 6B6, 6B7	97%, *H. armigera*	41374	4.08	3 > 1 > 6, male	Up
		6B31	77%, *S. littoralis*	2950	1.67	Male	Up
		6AB31	74%, *S. littoralis*	5881	2.39	1 > 3 > 6, pupa, Adults	Up
		6B6	99%, *H. armigera*	39825	2.09	6 > 3 > 1	Up
		6B2, 6B6, 6B7	89%, *H. armigera*	13679	4.38	3	Up
		6AN4	72%, *S. littoralis*	12093	1.96	Egg,female,1 > 3 > 6	Up
		6AN4	78%, *S. littoralis*	1833	1.07	1 < 3 < 6	Up
		6AB4	74%, *B. mori*	42286	5.24	Male	Up
		6AB14	68%, *S. littoralis*	15424	−1.39	3 > 6	Down
		6AE12	72%, *H. armigera*	41540	2.64	Female, male	Up
		6AE12	90%, *H. armigera*	3564	2.35	3 > 6 > 1, female	Up
		6AE14	99%, *H. armigera*	9094	−1.55	1 > 3 > 6	Down
		6AE14	78%, *H. armigera*	4567	1.68	1 > 3 > 6, female, male	Up
		6AE14	100%, *H. armigera*	15388	1.97	All	Up
		6AE14	92%, *H. armigera*	6041	1.16	3 > 6 > 1	Up
		6AE47	75%, *S. littoralis*	39097	2.37	6 > 1 > 3, female	Up
		6AE14	72%, *H. armigera*	30146	3.25	egg, 3 > 1 > 6, female	Up
		324A6	72%, *S. littoralis*	40289	2.84	3, pupa	Up
		337B3	99%, *H. armigera*	2773	2.64	Egg, female	Up
		337B3v1	85%, *H. armigera*	12899	1.29	All, 3 > 6 > 1	Up
		35D18	97%, *H. armigera*	17285	−3.63	6 > 3 > 1	Down
		35D18	91%, *H. armigera*	11744	−4.67	6 > 3 > 1	Down
		324A1	77%, *S. littoralis*	36658	2.03	Female, male	Up
		321A1	96%, *H. zea*	38041	4.82	Egg, 1 < 3 < 6	Up
		321A1	95%, *H. zea*	6465	6.56	3 > 6 > 1, male	Up
		321A2	91%, *H. zea*	9454	2.62	Egg, female	Up
		321A2	89%, *H. zea*	2372	1.53	Egg, pupa, female	Up
		321B1	90%, *S. littoralis*	40435	1.84	egg, female	Up
		9A18	88%, *H. armigera*	35600	−3.47	6 > 1 > 3, pupa	Down
		9A18	99%, *H. armigera*	40298	−3.49	All, 6 > 3 > 1	Down
		9A18	99%, *H. armigera*	6517	−4.46	6 > 1 > 3	Down
		9A12	*96%, H. armigera*	16315	1.511	3 > 1 > 6, female, male	Up
		9A14	94%, *H. zea*	13079	1.65	Egg, female	Up
		337B3v7	96%, *H. zea*	33786	1.50	Egg, female, 3 > 1 > 6	Up
		321A2	92%, *H. zea*	12163	7.21	6 > 3 > 1, male	Up
	CYP4	4 V2	88%, *Mamestra brassicae*	17185	2.32	All, 3 > 1 > 6	Up
		340 K4	69%, *S. littoralis*	8795	−1.95	3 > 1 > 6	Down
		4 M7	96%, *H. zea*	32914	3.18	3 > 6 > 1, pupa	Up
		4 M7	97%, *H. zea*	34657	1.79	3 > 6 > 1, pupa	Up
		4 L12	74%, *S. littoralis*	41937	5.61	All, 1 > 3 > 6	Up
		340AA1	66%, *S. littoralis*	18087	2.03	Egg, 3	Up
		340AA1	70%, *S. littoralis*	23572	1.03	3, male	Up
		4M14V1	76%, *S. litura*	22567	1.50	Pupa	Up
		4C1	71%, *Blaberus discoidalis*	26692	1.77	All	Up
		4S1	96%, *H. armigera*	1070	−1.29	6 > 1 > 3,female,pupa	Down
		367B6	73%, *S. littoralis*	15813	2.03	3, pupa, female	Up
		340AA1	70%, *S. littoralis*	21273	2.03	3	Up
		4G74	83%, *S. littoralis*	4194	−1.91	3 > 6 > 1,pupa, female	Down
		4G15	*D. melanogaster*	2239	−3.21	1 = 3 < 6, female	Down
		4G74	86%, *S. littoralis*	14395	−3.56	3 > 6 > 1, pupa, female	Down
		341B1	76%, *B. mori*	3370	−1.92	6 > 3 > 1	Down
		341B1	67%, *B. mori*	40986	−1.19	Pupa	Down
		341B3	78%, *S. littoralis*	7936	−1.14	6 > 3 > 1	Down
		341A13	83%, *S. littoralis*	26038	−1.55	3, female	Up
		4C1	88%, *B. mori*	22272	−2.43	6	Down

^a^Ratio: RPKM of 2-TD treated samples/RPKM of untreated samples. RPKM: Reads per kilo bases per million reads. RPKM < 0.1 was used as the criterion to judge the unigenes were not expressed during a given developmental stage. Absolute value of Log_2_Ratio ≥ 1 were used as thresholds for ‘differential expression’. The P450 genes reported to be involved in insect hormone biosynthesis and metabolism are shown in bold. 1: 1^st^ instar larvae; 3: 3^rd^ instar larvae; 6: 6^th^ instar larvae

^b^The cytochrome P450 clan schema used here follows the system proposed by Feyereisen *et al*. (2006)

### 2-TD-induced P450 genes related to hormone biosynthesis and metabolism

**Fig. 5:**
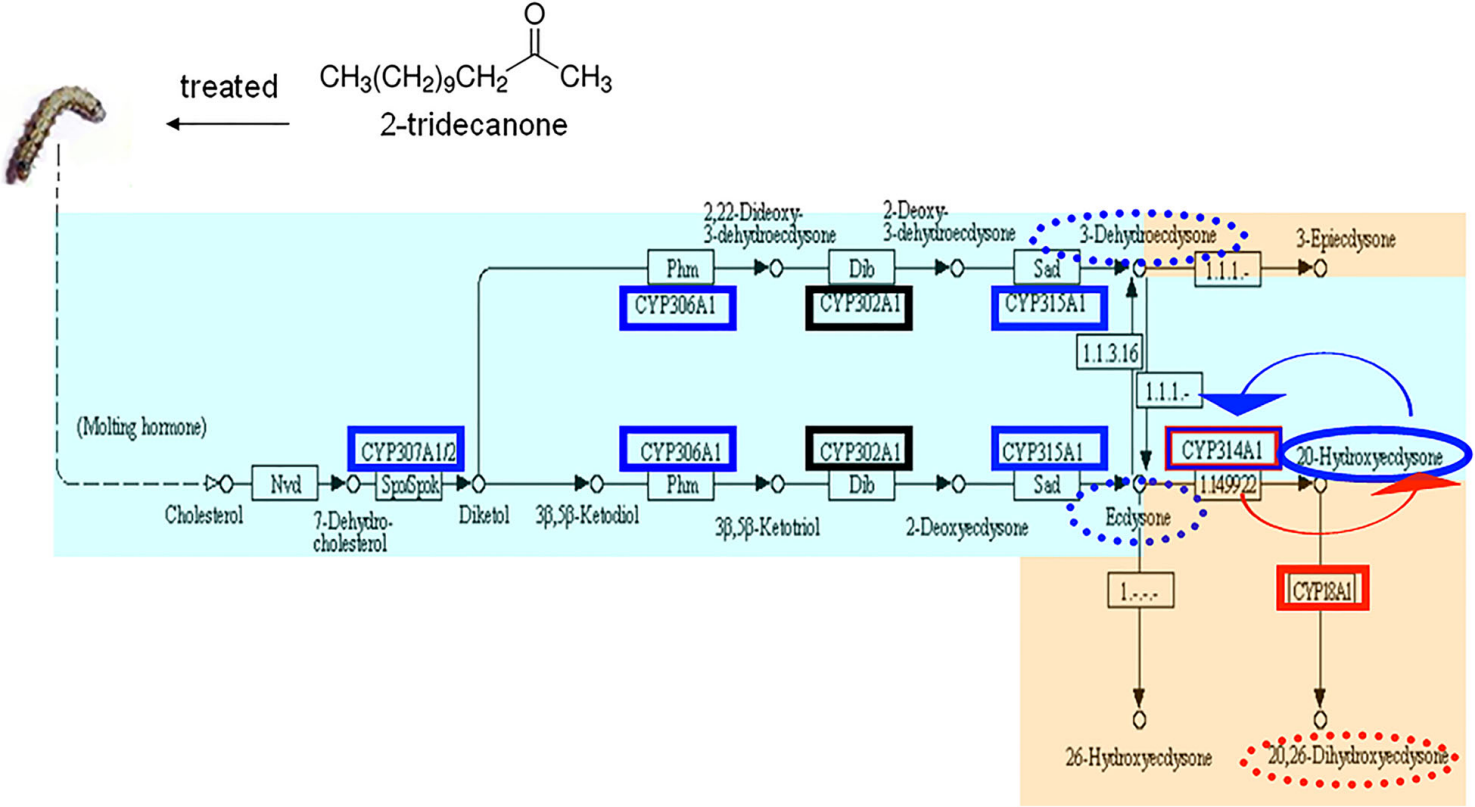
2-TD affects the biosynthesis and metabolism of insect hormones (JH and molting hormone). Genes down-regulated following treatment with 2-TD are shown with solid blue lines, up-regulated genes are shown with solid red lines. The dashed blue and red lines indicate the down- and up-regulated products, respectively. The portion with a blue background shows the biosynthetic pathway of 20E; portions with red backgrounds show the metabolic pathways of insect hormones [25, 29, 33, 34]

Pathway analysis of the unigenes was conducted using the Kyoto Encyclopedia of Genes and Genomes (KEGG) annotation system. To confirm the unigenes expression profile results, the expression of P450 unigenes induced by 2-TD that related to hormone metabolism was analyzed with Real-Time qPCR (Additional file 9). Figure 4 illustrates 2-TD affects the biosynthesis and metabolism of insect hormones (JH and molting hormone). The expression of four P450 are suppressed by 2-TD treatment: *CYP307A1* (unigenes 8199, 13311), *CYP306A1* (unigenes 13036), *CYP314A1* (unigenes 15409), and *CYP315A1* (unigenes 40026), these down-regulated genes are shown with solid blue lines in Fig. 4. The expression of two P450 are significantly increased by 2-TD treatment: *CYP18A1* (unigenes 41217, 35643, 16953), *CYP314A1* (unigenes 16487), these up-regulated genes are shown with solid red lines in Fig. 4. The dashed blue and red lines indicate the down- and up-regulated products, respectively, and Fig. 5 shown that 20E titers in the 2-TD treated group were higher than in the control group. The expression of three hormone metabolism related unigenes were not affected by 2-TD treatment (Additional file 9, Fig. 5). The Real-Time qPCR results of the other 2-TD-induced 22 P450 unigenes were consistent with the DGE gene expression profiles, suggesting that the DGE results were reliable (Additional file 10).

### RNA interference (RNAi) insect hormones-related P450 genes

**Fig. 6:**
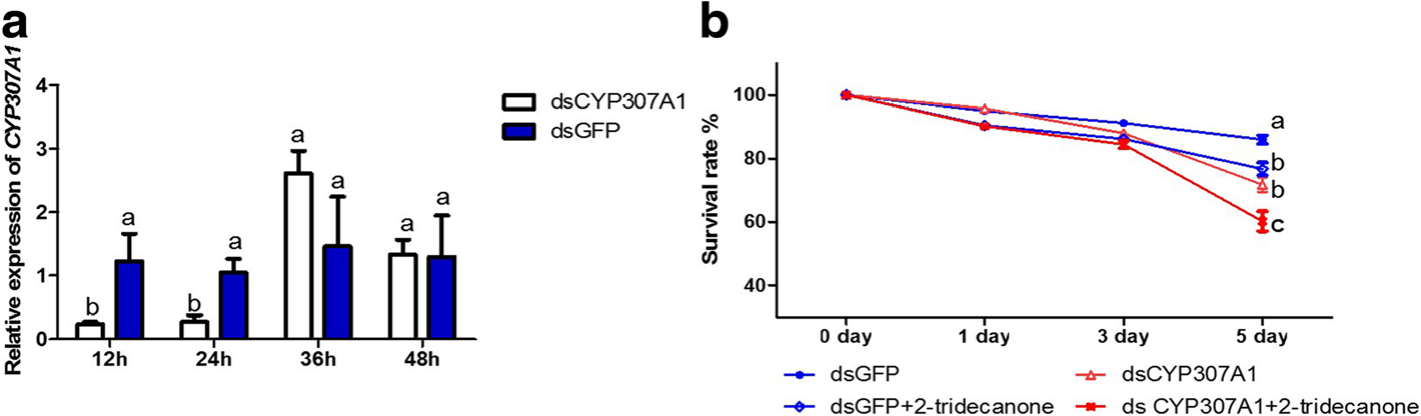
*CYP307A1* RNAi. **a** The dsRNA-mediated depletion of *CYP307A1* transcripts in larvae fed with *CYP307A1* dsRNA. **b** RNAi *CYP307A1* effects on the development of *H.armigera.* Second-instar larvae were fed on a diet containing 5 μg/g or 35 μg/g (w:w) dsRNA, and samples were collected at 12, 24, 36, and 48 h after feeding. *GFP* dsRNA was used as a control, at the same concentrations. In the each diagram, bars sharing the same letter for each time point group are not significantly different at the *P* >0.05 level

*CYP307A1* (unigenes 8199, 13311), an insect hormone-related P450 gene that was down-regulated by 2-TD in *H. armigera*, was selected for RNAi knockdown studies. The *CYP307A1* dsRNA-treated larvae showed significant reduction of *CYP307A1* expression as compared to the larvae treated with *GFP* dsRNA (Fig. 6a). Compared to the control, 90 and 85% of *CYP307A1* expression was suppressed at 12 h and 24 h after feeding larvae artificial diet with 35 μg/g (w:w) *CYP307A1* dsRNA, respectively. However, no significant retardation of transcription was observed at 36 or 48 h after feeding (Fig. 6a).

The effect of the RNAi-based knockdown of *CYP307A1* on larval survival rates was evaluated in second instar larvae by feeding artificial diet mixed with 35 μg/g (w:w) *CYP307A1* dsRNA and 2-TD (0.1 mg/g) for 1, 3, and 5 days. Compared to treated with ds *CYP307A1* larvae, the survival rate dramatically decreased in larvae treated the mixture of *CYP307A1* with 2-TD. The survival rate was 72% for the treatment with ds *CYP307A1* and 61% for the mixture of *CYP307A1* with 2-TD, through continuous feeding for 5 days (Fig. 6b).

## Discussion

Our experimental results showed that the plant allelochemical 2-TD affects insect development (Table 1), and we observed that a decrease in the concentration of 20E occurred along with the growth retardation symptoms following 2-TD treatment (Table 1). 2-TD treatment induced the expression of P450 detoxification enzyme genes. Insect P450 enzymes are classified into four major clans, namely the CYP2, CYP3 (including CYP6 and CYP9), CYP4, and the mito.CYP clan [23]. The mito.CYP, CYP2, and CYP4 clans contain a variety of single-member CYP families that are known to play important roles in diverse physiological processes [24–31]*.* The CYP3 clan in insects can be further subdivided into the CYP6 and CYP9 families, which participate primarily in the metabolism of xenobiotic compounds [19, 32].

20E is a polyhydroxylated steroid hormone that controls molting and thereby affects the growth of arthropods. Studies using *D. melanogaster* have revealed that the Halloween P450 genes (*CYP307A1*/*A2*, *CYP306A1*, *CYP302A1*, *CYP315A1,* and *CYP314A1*) are essential for each of the steps in 20E biosynthesis [25, 33, 34]. *CYP18A1* belongs to the CYP2 clan and takes part in 20E inactivation, converting 20E to 20, 26-dihydroxyecdysone [29]. In *B. mori, CYP18A1* not only has temporal- and tissue-specific expression profiles, but also exhibits a distinct expression pattern that closely coincided with the peak of ecdysteroid accumulation in the hemolymph of *B. mori*, a finding that further suggests that orthologous *CYP18A1* in insects is closely related to ecdysteroid homeostasis [35]. Interestingly, we also observed that 2-TD treatment dramatically increased the expression of *CYP18A1* (41217, 35643, 16953) (Table 3, Fig. 5), the increasing *CYP18A1* will lead a lower concentration of 20E. Our results clearly show that treatment of larvae with 2-TD decreased 20E concentrations (Table 1) and suppressed larval growth. *CYP306A1* (13036), *CYP307A1* (8199, 13311)*, CYP314A1* (16487, 15409), and *CYP315A1* (40026) may be also essential for 20E biosynthesis. Treatment with 2-TD decreased the expression levels of these unigenes. We used RNAi methods to confirm the function of *CYP307A1* in *H. armigera*. Larvae treated with *CYP307A1* dsRNA had dramatically decreased survival rates compared to the *GFP* dsRNA control, this symptom was similar with RNAi *CYP307A1* in *D. melanogaster* [36]. Compared to treated with ds *CYP307A1* larvae, the survival rate dramatically decreased in larvae treated the mixture of *CYP307A1* with 2-TD (Fig. 6b), these results proved that the retardant effect of 2-TD is mediated by *CYP307A1* on development of cotton bollworm. Some unigenes were annotated as the same P450 gene, but these unigenes have different expression profiles in one sample, these phenomenon maybe caused by these unigenes are not full-length P450 genes or they have allele genes.

*CYP15A1* encodes an enzyme that catalyzes the last step in JH biosynthesis, catalyzing the epoxidation of methyl farnesoate into JH III in *D. punctate* [37]. *CYP4C7,* expressed in a heterologous system, was able to metabolize JH III and JH precursors into 12-transhydroxy [30]. Although *CYP4C7* and *CYP15A1* were not found to be regulated by 2-TD, the percentage of expressed CYP4 genes decreased following 2-TD treatment in *H. armigera* (Table 3), and the percentage of expressed CYP4 unigenes suddenly increased during the transformation from eggs to larvae; the percentage of expressed CYP4 decreased during the transformation from larvae to pupae (Fig. 1). 48 P450 unigenes of the CYP4 clan were found in our study. *CYP4C15*, initially cloned from the steroidogenic glands (Y-organs) of crayfish, has been suggested to be involved in ecdysteroid biosynthesis [38]. In *Diploptera punctata*, *CYP4C7* is expressed selectively in the corpora allata and metabolizes JH and its precursors into new metabolites [10, 30]. CYP4 unigenes in *H. armigera* homologous to *CYP15A1* and *CYP4C7* may be involved in JH biosynthesis and metabolism. In our study, the expression of these genes in larvae was higher than in eggs, and was induced by 2-TD treatment (Table 3). Four CYP4 unigenes (12812, 10466, 10601, 14820) were specifically expressed in larvae, and one CYP4 unigene (22936) was solely expressed in females. These expressed P450 unigenes seem likely to play important roles during these specific stages (Table 1). 2-TD treatment strongly induced the expression of CYP4 unigenes in 6^th^ instar larvae (Fig. 4a), a finding consistent with previous research in other insects [23]. These imply that the increased expression of CYP4 transcripts induced by 2-TD treatment would likely also affect JH biosynthesis and metabolism.

Our DGE analysis found that *CYP333B3* (12317) and *CYP333A3* (25319), which belong to the mito.CYP clan, were also regulated by 2-TD (Table 3). Other 2-TD-regulated mito.CYP genes are related to the metabolism of molting hormone, but there have been no reports to prove that these two unigenes are involved in the biosynthesis or metabolism of molting hormone. Both the up- and down-regulation of these two P450 unigenes may be of critical importance in the development and metamorphosis of insects. As many of these genes are conserved among many insect species, our study provides a foundation for the functional characterization of the roles of these two P450 unigenes in insect development and metamorphosis.

About 80 P450 unigenes of the CYP3 clan were identified in our study. Within the genus Papilio (Lepidoptera: Papilionidae), CYP6 family members are known to detoxify furanocoumarins, secondary metabolites characteristic of the host plant families consumed by these insects [14, 39–45]. In our study, four P450 unigenes (13679, 22278, 15388, 19180) shared homology with *CYP6B2*, *CYP321B1*, *CYP6AE14,* and *CYP9A18*. These unigenes all belong to the CYP3 clan are known to be specifically expressed in larvae, and are thought to participate primarily in the metabolism of plant allelochemicals [12, 14]. Two CYP3 unigenes were expressed only in adult females. One CYP3 unigene was specifically expressed in egg and pupa, respectively (Table 2). The ability of insects to metabolize xenobiotic compounds at different development stages may be related to these CYP3 clan P450 unigenes.

## Conclusions

In conclusion, we found that 2-TD can retard the development of cotton bollworm, and a decrease of the concentration of 20E occurred alongside the retardant symptoms (Table 1). In order to further illuminate the relationship between 2-TD and its function in retarding the development of insects, the transcriptome of *H. armigera* was sequenced and digital gene expression libraries were constructed in the present study. The expression of *CYP314A1*, *CYP315A1*, *CYP18A1*, *CYP307A1,* and *CYP306A1* was found to be induced by 2-TD, and these genes were also related to the biosynthesis or metabolism of 20E. Expression analysis and RNAi studies proved that the retardant effect of 2-TD is mediated by P450 genes on development of cotton bollworm.

## Methods

### Insect samples

The cotton bollworm population used in this study (a laboratory population) was initially collected from the Handan region of Hebei Province, China, in 1998, and reared on an artificial diet in a growth room maintained at 26 ± 1 °C, 70–80% relative humidity, with a photoperiod of 16:8 (L:D). The population was never exposed to any pesticides. The composition of the artificial diet was as follows: corn flour 300 g, soybean powder 100 g, yeast extract powder 100 g, citric acid 2.5 g, vitamin C 10 g, sorbic acid 1.5 g, vitamin B 1.5 g, erythromycin 0.05 g, propionic acid 5 mL, vitamin E 1.5 g, water 2.5 L. Adults were held under the same conditions and supplied with a 10% sugar solution. Females were induced to oviposit into gauze. Eggs were collected from this gauze. All specimens at all life stages were pesticide-free and were reared in a growth chamber set to the aforementioned environmental conditions. The newly molted 6^th^ instar larvae, after molted for 1 day, were treated by 12 h of starvation treatment, then the larvae were exposed to the artificial diet mixed with 2-TD (Sigma-Aldrich, MO, USA) (99% purity) 10 mg/g (w:w) for 24 h (ethyl alcohol as negative control). Each treatment contained twenty five larvae, and these experiments were repeated four times.

### Quantification of Ecdysteroids

Total 20Ewere quantified by enzyme immunoassay (EIA). Newly molted sixth instar larvae treated with 2-TD (twenty five larvae/tube with four replicates) were homogenized and extracted as described previously [46]. The extracts were evaporated, redissolved, and subjected to ecdysteroid enzyme-linked immunosorbent assay (ELISA). The ELISA was performed in a competitive assay format using anti-20E rabbit antiserum (Cayman Chemical, Michigan, USA), acetylcholinesterase-conjugated 20E (Cayman Chemical, Michigan, USA), and standard 20E (Sigma-Aldrich, St. Louis, MO, USA). The acetylcholinesterase activity was quantified by Ellman’s Reagent (Cayman Chemical, Michigan, USA), and the absorbance at 415 nm was detected with a Benchmark microplate reader (Bio-Rad Laboratories, Hercules, USA).

### RNA isolation

Total RNA was isolated from specimens at the following developmental stages: eggs collected within 24 h of post-oviposition; first-instar larvae; third-instar larvae; sixth-instar larvae not treated with 2-tridecane; pupae; mating adults (females and males, within 6 days of eclosion); and sixth-instar larvae treated with 2-TD. For each sample, approximately 800 mg of insect material was homogenized with liquid nitrogen in a mortar in order to reduce the effect of error. RNA was extracted using an RNeasy plus Micro Kit (Qiagen GmbH, Germany) following the manufacturer’s instructions. RNA was quantified by measuring the absorbance at 260 nm using a NanoDrop® 1000A spectrophotometer (GE Healthcare, Uppsala, Sweden). The purity of all RNA samples was assessed at an absorbance ratio of OD_260/280_ and OD _260/230_, and the integrity of RNA was confirmed by electrophoresis on 1% agarose gels.

### Construction of the cDNA library and Illumina sequencing for transcriptome analysis

Briefly, 12 mg total RNA (a mixture of RNA from eggs, 1^st^ instar larvae, 3^rd^ instar larvae, 6^th^ instar larvae, pupae, adult females and males, all at equal proportions) was used to construct a cDNA library of transcriptome. Poly (A) mRNA was purified from total RNA using oligo (dT) magnetic beads. These short fragments were then used as templates for the synthesis of first-strand cDNA. Second-strand cDNA was synthesized using DNA polymerase I, and the samples were treated with RNaseH. Short fragments were purified using a QiaQuick PCR extraction kit (Qiagen GmbH, Hilden, Germany). These fragments were subsequently washed with elution buffer for end reparation poly (A) addition and then ligated to sequencing adapters. Suitable fragments, as determined by agarose gel electrophoresis, were selected for use as templates for PCR amplification. The cDNA library was sequenced using the Illumina Solexa platform.

### Assembly and functional annotation of the transcriptome

Using Trinity program to assembly transcripts, all of the raw sequences were filtered to remove low quality and adaptor sequences [47]. Open reading frame (ORF) of the unigenes were predicted using the ORF finder tool (https://www.ncbi.nlm.nih.gov/orffinder/). All unigenes were queried against the NCBI Nr protein database with an e-value cutoff of 1e^−5^ for functional annotation. The BLASTN algorithm was also used to query the unigenes against the NCBI Nt nucleotide databases (Nt; e-value < 10^-5^). For quantitative assessment of the assembly and annotation completeness, in comparison with the arthropod profile in OrthoDB v8 [48], we applied the software tool BUSCO [49], which is based on evolutionarily informed expectations of gene content, with default settings. Then, the BLAST results were used to do a tentative functional annotation of the unigenes with GO, KEGG and COG databases (e-value < 10^-5^). The clean reads and computationally assembled sequences from this study were submitted to the Sequence Read Archive (SRA) database (Accession number: SRX374716).

### Selection of cytochrome P450 sequences and phylogenetic analysis

Sequences encoding genes related to cytochrome P450s were identified by BLASTX analysis against the NCBI nr database, with a cut-off value of e-value < 10^-5^. Sequences that returned redundant BLASTX results, or those that showed high homology with each other as determined by the alignment results, were eliminated as likely allelic variants or different portions of the same gene. MEGA 6.0 software was used to analyze the phylogenetic relationships between the P450 unigenes of *H. armigera* and the published P450 sequences from *Bombyx mori* (*B. mori*). The amino acid sequences for each predicted protein were aligned using MAFFT 7.110 [50]. Neighbor-joining trees were produced using MEGA 6.0 with Poisson correction of distances [51], and 1000 neighbor-joining bootstrap replicates.

### Preparation and sequencing of the DGE library

RNA was extracted separately from eggs, 1^st^ instar larvae, 3^rd^ instar larvae, 6^th^ instar larvae, pupae, adults (females and males), and sixth-instar larvae treated with 2-TD. The extractions were performed using an RNeasy plus Micro kit (Qiagen GmbH, Hilden, Germany) according to the manufacturer’s instructions. Approximately 10 μg RNA from each sample was used for the construction of DGE libraries. mRNA was treated as described in the cDNA library construction methods, above. The fragments were purified by agarose gel electrophoresis and enriched by PCR amplification. The library products were then sequenced with the Illumina Solexa platform. The raw data (tag sequences and counts) were deposited in the NCBI SRA database, under accession number: SRX684363.

### Bioinformatics pipeline and analysis of DGE libraries

Sequencing raw data were transformed by base calling into raw sequence data. Clean tags were obtained after the raw sequences were filtered to remove adaptor sequences, empty tags, low quality tags, tags that were too short (<200 bp), and tags with a copy number of 1. All clean tags were mapped to the transcriptome of *H. armigera* with a stringency allowing no more than 1 nucleotide mismatch. The number of unambiguous, clean tags for each gene was calculated, and then normalized to RPKM (Reads Per Kilo bases per Million reads), using the following equation: $RPKM=\frac{10^6/\mathrm{C}}{NL/{10}^3}$ in which *C* is the number of reads uniquely mapped to a given gene, *N* is the number of reads uniquely mapped to all genes, and *L* is the total length of the exons in the given gene. For genes with more than one alternative transcript, the longest transcript was selected to calculate the RPKM. The RPKM method eliminates the influences of different gene lengths and sequencing discrepancies on gene expression calculations. Therefore, RPKM values can be used directly for comparing differences in gene expression among samples. [52]. RPKM <0.1 was used as the criterion to judge if a given unigene was not expressed in one specimen.

For gene expression profiling analysis, unigenes were assigned GO terms using the Blast2GO and canonical pathways tools of the KEGG pathway enrichment analysis. Analysis of the differentially expressed genes was performed based on the GOstat algorithm [53]. To identify the differentially expressed genes among different development libraries (egg, 1^st^ instar larvae, 3^rd^ instar larvae, 6^th^ instar larvae, pupae, adult females and males libraries), each library compared with egg library, and the fold change Log_2_ Ratio ≥ 1 values were used as threshold criteria to judge the differences in gene expression [54]. Compared with 6^th^ instar larvae library, the differentially expressed genes among 6^th^ instar larvae library and 2-TD treated library were also identified by Log_2_ Ratio ≥ 1 values. The percentage of each CYP clan (mitochondrial, clan 2, clan3 and clan4) expressed in each DGE library was calculated according to the following formula: (Sum RPKM of each CYP clan)/(Sum RPKM of P450) × 100%.

### Validation of P450 gene expression profiles by Real-Time PCR

To confirm the gene expression profile results from the DGE libraries, the expression of 35 P450 unigenes (including 12 hormone-related P450 unigenes) were analyzed with Real-Time qPCR. Specific primers were designed using Primer 5.0 software, and are listed in Additional file 12. *EF-α* was used as an internal control. Three biological replicates were performed for qPCR assay. The efficiency of each set primer was about 100% (Additional file 12). RNA isolation was performed using TRIzol reagent, according to the manufacturer’s instructions (Invitrogen, Carlsbad, CA, USA). Samples were treated with RNase-free DNase I (Takara Biotechnology Dalian Co., Ltd., Dalian, China). First-strand cDNA synthesis was performed with 1 μg of total RNA by using a Transcriptor First Strand cDNA Synthesis Kit (Takara Biotechnology Dalian Co., Ltd., Dalian, China). cDNA was amplified using an Applied Biosystems7500 qPCR System (Applied Biosystems, Foster City, USA) with a Real Master Mix SYBR Green PCR kit (Invitrogen Carlsbad, CA, USA). Amplification conditions consisted of an initial pre-incubation at 95 °C for 5 min, followed by amplification of the target DNA for 40 cycles of 94 °C for 30 s, 60 °C for 30 s, 72 °C for 30s and 95 °C for 5 min. The melting curves of the amplicons were measured by taking continuous fluorescence readings whilst increasing the temperature from 58 to 95 °C, with 0.5 °C incremental increases every 10 s. geNorm version 3.5 [55] and Normfinder version 0.953 [56] software were used to evaluated the raw CT values of the selected reference genes as described in their manuals. Candidate gene with the lowest M value should be the most stably expressed reference gene, and *EF-1a* was chose as the reference gene (Additional file 13). For each gene, a standard curve was generated for each set of primers, and the efficiency of each reaction was determined.

Statistical analyses of Real-Time qPCR results were performed using GraphPad Prism 5.0 software (GraphPad prism, Prism 5 for Windows). Statistical significance was determined by using a Student’s t-test, and a p value less than 0.05 was considered to indicate statistical significance.

### RNAi insect hormone-related P450 genes

Based on the *CYP307A1* gene sequence (Gene bank number: KM016704.1) and predicted possible interference sites obtained using online prediction software (http://www.dkfz.de/signaling/e-rnai3/), we designed specific primers using DNAMAN 6.0 software. A 494-bp fragment of *CYP307A1* (position 730–1310) was amplified and cloned into the pMD-18simple-T vector (Takara, Dalian, China), using the dsRNAi-CYP307A1-1 and dsRNAi-CYP307A2-2 primer pair (Additional file 12), which contained additional T7 promoter sequences. Purified plasmids served as templates for RNA synthesis using a MEGAscript T7 transcription kit (Ambion, Austin, TX, USA). *GFP* dsRNA, which was used as the control, was synthesized with the same procedures as above, using the dsGFP-F and dsGFP-R primers (Additional file 12). dsRNA from GFP and *CYP307A1* were derived by using the MEGAscript T7 transcription kit with an extended transcription time of 5 h at 37 °C. The resulting dsRNA was digested by DNase I and RNase to remove DNA and any single-stranded RNA, and finally dissolved in DEPC water.

Second-instar larvae, after being starved for 12 h, were exposed to artificial diet containing *CYP307A1* dsRNA (15 μg/g or 35 μg/g, w/w) mix or not mix with 2-TD (0.1 mg/g, w/w) for 12, 24, and 36 h; *GFP* dsRNA was used as a control. Thirty larvae were used in each treatment, and three replications were performed. The dsRNA-mediated depletion of *CYP307A1* transcripts was experimentally evaluated with qPCR by using the q*CYP307A1* -F and q*CYP307A1* -R primers (Additional file 12).

## Additional files

Additional file 1: Figure 6^th^ instar larvae treated with 2-TD (10 mg/g). (PDF 170 kb)
Additional file 2: Output statistics of sequencing and assembly quality for the transcriptomes of *H. armigera*. (DOCX 13 kb)
Additional file 3: Species distribution of the BLASTX results. This figure shows the species distribution of unigene BLASTX matches against the nr protein database (cutoff value e < 10^-5^). (PDF 214 kb)
Additional file 4: BUSCO analysis results. (XLSX 79 kb)
Additional file 5: COG function classification of consensus for the transcriptome of *H. armigera*. (PDF 68 kb)
Additional file 6: Classification of the gene ontology (GO) for the transcriptome of *H. armigera*. Transcripts were annotated in three categories: cellular components, molecular function and biological processes. (PDF 81 kb)
Additional file 7: Unrooted distance neighbor-joining tree of P450 sequences from *H. armigera* (Red) and *B. mori* (Black). Homology with greater than 70% support with 1000 bootstrap replications is indicated at the corresponding nodes. Branches in different colors show different clans (the mito.CYP clan is shown in red, CYP2 in green, CYP3 in yellow, and CYP4 in blue). (PDF 326 kb)
Additional file 8: Comparison of the number of digital tags generated from the different stages of *H. armigera*. (DOCX 14 kb)
Additional file 9: Validation of hormone-regulated P450 genes expression by Real Time qPCR. (A) hormone-regulated P450 genes which were down-regulated by 2-TD. (B) hormone-regulated P450 genes which were up-regulated by 2-TD. (C) hormone-regulated P450 genes which were not significant inducible by 2-TD. (PDF 398 kb)
Additional file 10: Validation of gene expression by RT-PCR. (A) P450 unigenes which were regulated by 2-TD. (B) P450 unigenes which were not significantly induced by 2-TD. (C) P450 unigenes expressed at specific Developmental stages. (PDF 563 kb)
Additional file 11: The expression of the 153 P450 unigenes in different DGE library. (XLSX 25 kb)
Additional file 12: Primers were used in this manuscript. (DOCX 18 kb)
Additional file 13: Expression stability of the candidate reference genes under different conditions. (DOCX 13 kb)

## Abbreviations

2-TD: 2-Tridecanone
BLASTX: Similarity search of the NCBI protein database using a translated nucleotide query
COG: Clusters of orthologous groups
DGE: Digital gene expression
EIA: Enzyme immunoassay
ELISA: Enzyme-linked immunosorbent assay
GO: Gene ontology
JH: Juvenile hormone
KEGG: Kyoto Encyclopedia of Genes and Genomes
nr: NCBI non-redundant protein sequences database
nt: NCBI nucleotide collection
ORF: Open reading frame
RNAi: RNA interference
RPKM: Reads Per Kilo bases per Million reads
SRA: Sequence Read Archive
