# Supplementary material for: The retardant effect of 2-Tridecanone, mediated by Cytochrome P450, on the Development of Cotton bollworm, Helicoverpa armigera
Source: BMC Genomics. 2016 Nov 22;17:954. doi: 10.1186/s12864-016-3277-y (PMC5118896; doi:10.1186/s12864-016-3277-y)
Supplement: Additional file 1: — Figure 6th instar larvae treated with 2-TD (10 mg/g). (PDF 170 kb) [file 12864_2016_3277_MOESM1_ESM.pdf]

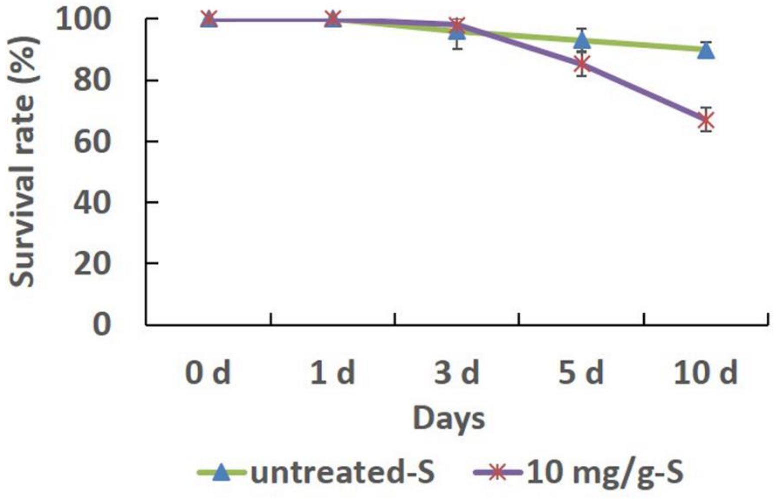

Figure 6th instar larvae treated with 2-TD (10 mg/g). untreated-S: show the survival rate of the untreated larvae;

10 mg/g-S: show the survival rate of the larvae treated with 10 mg/g 2-TD.
