# Supplementary material for: The retardant effect of 2-Tridecanone, mediated by Cytochrome P450, on the Development of Cotton bollworm, Helicoverpa armigera
Source: BMC Genomics. 2016 Nov 22;17:954. doi: 10.1186/s12864-016-3277-y (PMC5118896; doi:10.1186/s12864-016-3277-y)
Supplement: Additional file 2: — Output statistics of sequencing and assembly quality for the transcriptomes of H. armigera. (DOCX 13 kb) [file 12864_2016_3277_MOESM2_ESM.docx]

**Table Output statistics of sequencing and assembly quality for the transcriptomes of *Helicoverpa armigera***

| Total Clean Reads | 43, 756, 144 |
| --- | --- |
| Total nucleotides | 4, 419, 370, 544 |
| Total number of Transcripts | 93, 896 |
| Total number of unigenes | 42, 463 |
| Q20 | 98.07 |
| >2000bp | 1, 145 |
| N50 length transcriots  N50 length unigenes | 597 bp  695 bp |
