# Supplementary material for: The retardant effect of 2-Tridecanone, mediated by Cytochrome P450, on the Development of Cotton bollworm, Helicoverpa armigera
Source: BMC Genomics. 2016 Nov 22;17:954. doi: 10.1186/s12864-016-3277-y (PMC5118896; doi:10.1186/s12864-016-3277-y)
Supplement: Additional file 3: — Species distribution of the BLASTX results. This figure shows the species distribution of unigene BLASTX matches against the nr protein database (cutoff value e < 10-5). (PDF 214 kb) [file 12864_2016_3277_MOESM3_ESM.pdf]

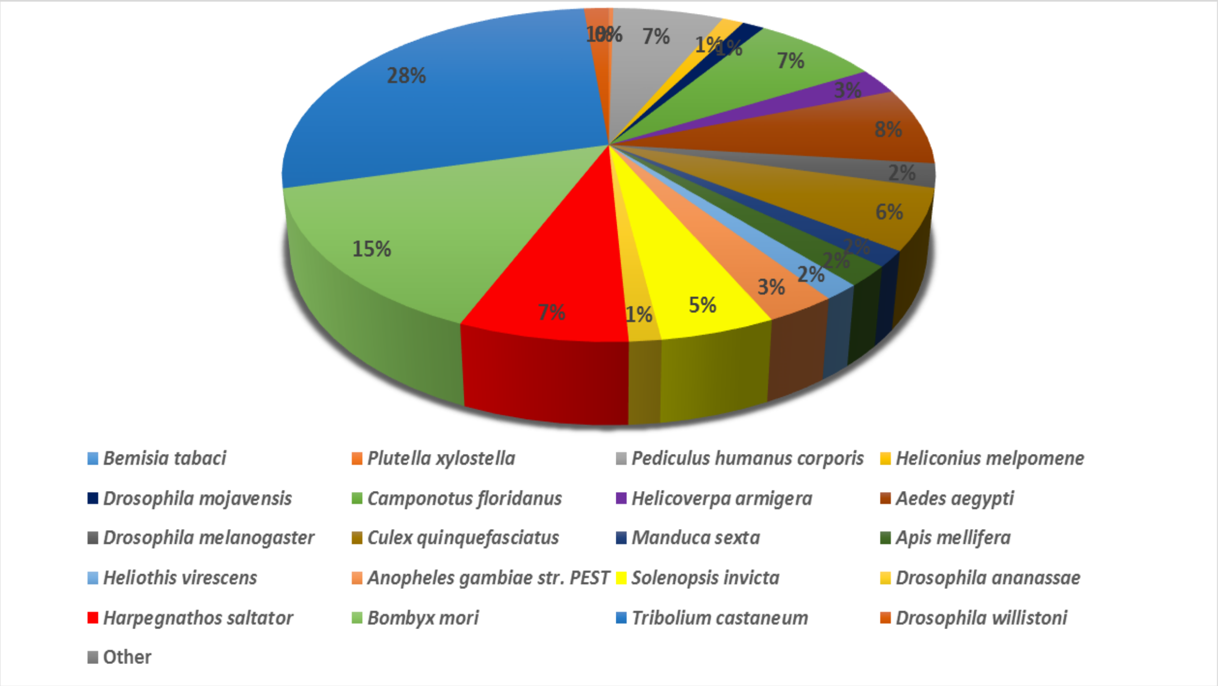

Fig. S1 Species distribution of the BLASTX results. This figure shows the species distribution of unigene BLASTX matches against the nr protein database (cutoff value $e<10^{-5}$).
