# Supplementary figures and images for: The retardant effect of 2-Tridecanone, mediated by Cytochrome P450, on the Development of Cotton bollworm, Helicoverpa armigera
Source: BMC Genomics. 2016 Nov 22;17:954. doi: 10.1186/s12864-016-3277-y (PMC5118896; doi:10.1186/s12864-016-3277-y)

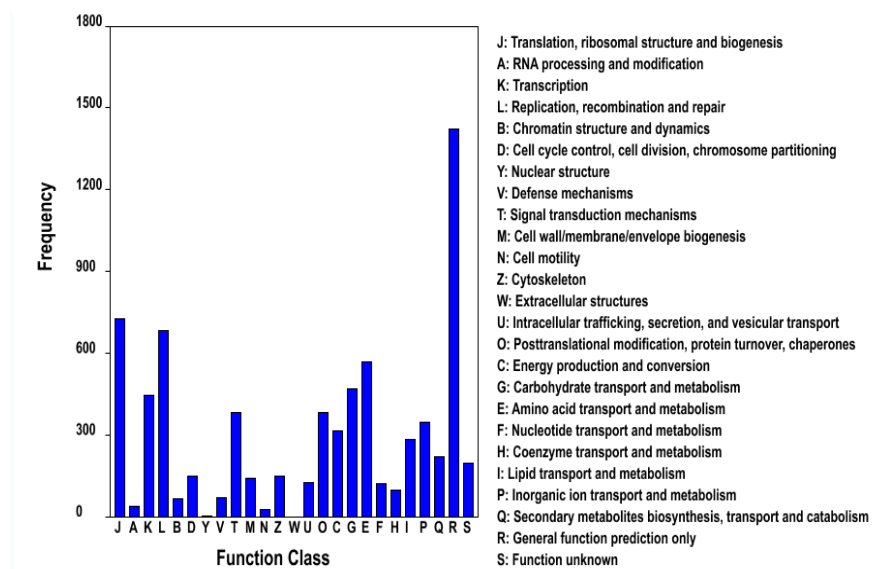

**Fig. S2 COG function classification of consensus for the transcriptome of *H. armigera*.**

Supplement: Additional file 5: — COG function classification of consensus for the transcriptome of H. armigera. (PDF 68 kb) [file 12864_2016_3277_MOESM5_ESM.pdf]
