# Supplementary material for: The retardant effect of 2-Tridecanone, mediated by Cytochrome P450, on the Development of Cotton bollworm, Helicoverpa armigera
Source: BMC Genomics. 2016 Nov 22;17:954. doi: 10.1186/s12864-016-3277-y (PMC5118896; doi:10.1186/s12864-016-3277-y)
Supplement: Additional file 6: — Classification of the gene ontology (GO) for the transcriptome of H. armigera. Transcripts were annotated in three categories: cellular components, molecular function and biological processes. (PDF 81 kb) [file 12864_2016_3277_MOESM6_ESM.pdf]

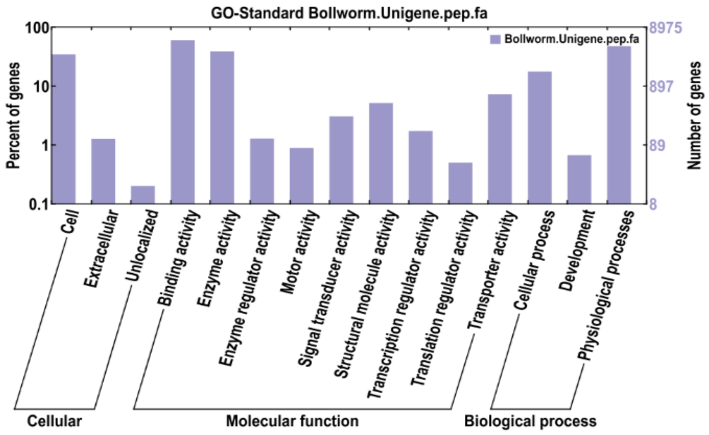

Fig. S3 Classification of the gene ontology (GO) for the transcriptome of *H. armigera*.

Transcripts were annotated in three categories: cellular components, molecular function and biological processes.
