# Supplementary material for: The retardant effect of 2-Tridecanone, mediated by Cytochrome P450, on the Development of Cotton bollworm, Helicoverpa armigera
Source: BMC Genomics. 2016 Nov 22;17:954. doi: 10.1186/s12864-016-3277-y (PMC5118896; doi:10.1186/s12864-016-3277-y)
Supplement: Additional file 7: — Unrooted distance neighbor-joining tree of P450 sequences from H. armigera (Red) and B. mori (Black). Homology with greater than 70% support with 1000 bootstrap replications is indicated at the corresponding nodes. Branches in different colors show different clans (the mito.CYP clan is shown in red, CYP2 in green, CYP3 in yellow, and CYP4 in blue). (PDF 326 kb) [file 12864_2016_3277_MOESM7_ESM.pdf]

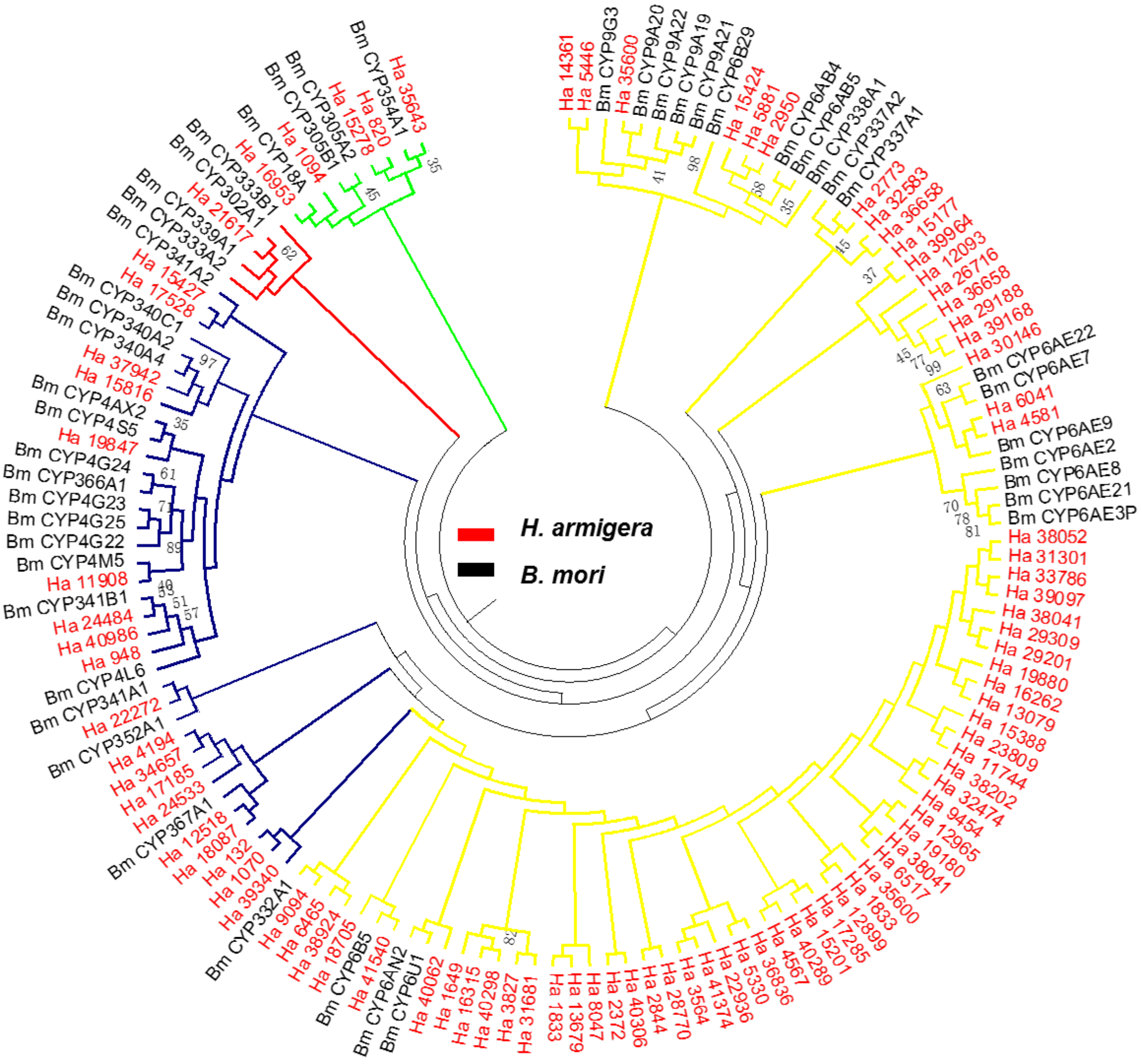

Fig. S4 Unrooted distance neighbor-joining tree of P450 sequences from *H. armigera* (Red) and *B. mori* (Black). Homology with greater than 70% support with 1000 bootstrap replications is indicated at the corresponding nodes. Branches in different colors show different clans (the mito. CYP clan is shown in red, CYP2 in green, CYP3 in yellow, and CYP4 in blue).
