# Supplementary material for: The retardant effect of 2-Tridecanone, mediated by Cytochrome P450, on the Development of Cotton bollworm, Helicoverpa armigera
Source: BMC Genomics. 2016 Nov 22;17:954. doi: 10.1186/s12864-016-3277-y (PMC5118896; doi:10.1186/s12864-016-3277-y)
Supplement: Additional file 8: — Comparison of the number of digital tags generated from the different stages of H. armigera. (DOCX 14 kb) [file 12864_2016_3277_MOESM8_ESM.docx]

**Table comparison of the number of digital tags generated from the different stages of *H. armigera***

| Summary | Egg | Larva | | | | Pupa | Adult | |
| --- | --- | --- | --- | --- | --- | --- | --- | --- |
|  |  | 1^st^ 3 ^rd^ 6 ^th^ Treated | | | |  | Female Male | |
| Total reads | 8,313,836 | 7,895,963 | 8,449,993 | 7,940,464 | 9,215,582 | 10,144,310 | 8,502,891 | 8,472,778 |
| Mapped reads | 5,884,850 | 5,965,999 | 6,404,067 | 6,431,874 | 6,707,863 | 7,464,216 | 6,032,737 | 7,207,974 |
| Perfect match | 4,133,696 | 4,085,926 | 4,289,229 | 4,824,015 | 4,368,136 | 4,973,462 | 4,282,421 | 5,696,514 |
| Unique match | 2,604,059 | 2,348,937 | 2,390,082 | 3,350,219 | 2,239,637 | 2,671,006 | 2,751,098 | 4,282,572 |
| ≤2bp mismatch | 1,529,637 | 1,736,989 | 1,899,147 | 1,473,796 | 2,128,499 | 2,302,456 | 1,531,323 | 1,413,942 |
| Q20 | 97.54 | 85.57 | 97.29 | 86.76 | 85.64 | 86.07 | 97.56 | 86.74 |

1^th^: first-instar larvae; 3 ^th^: third-instar larvae; 6 ^th^: sixth-instar larvae. Treated: sixth-instar larvae treated by 2-tridecaone for 24 hours.
