# Supplementary material for: The retardant effect of 2-Tridecanone, mediated by Cytochrome P450, on the Development of Cotton bollworm, Helicoverpa armigera
Source: BMC Genomics. 2016 Nov 22;17:954. doi: 10.1186/s12864-016-3277-y (PMC5118896; doi:10.1186/s12864-016-3277-y)
Supplement: Additional file 9: — Validation of hormone-regulated P450 genes expression by Real Time qPCR. (A) hormone-regulated P450 genes which were down-regulated by 2-TD. (B) hormone-regulated P450 genes which were up-regulated by 2-TD. (C) hormone-regulated P450 genes which were not significant inducible by 2-TD. (PDF 398 kb) [file 12864_2016_3277_MOESM9_ESM.pdf]

A: Down-regulated

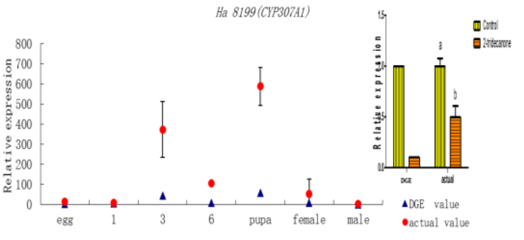

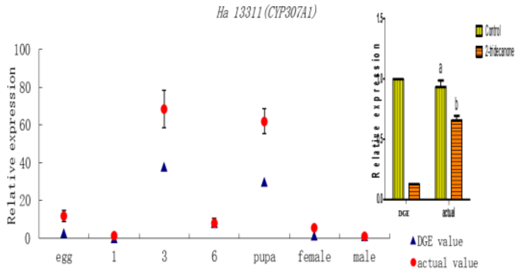

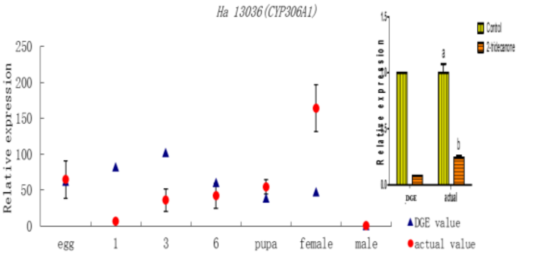

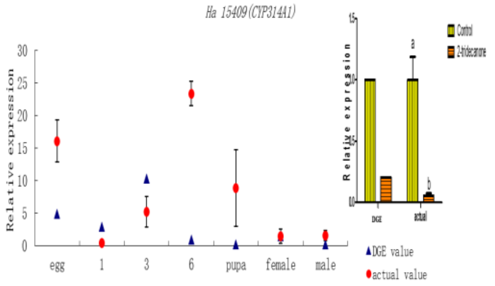

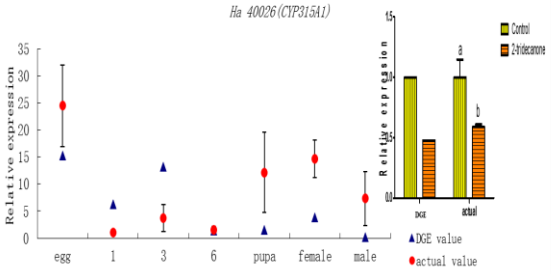

B: Up-regulated

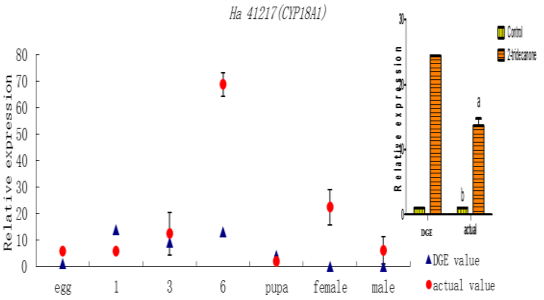

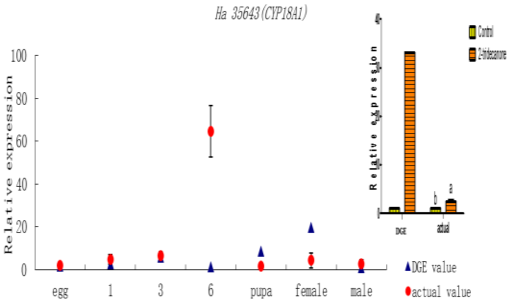

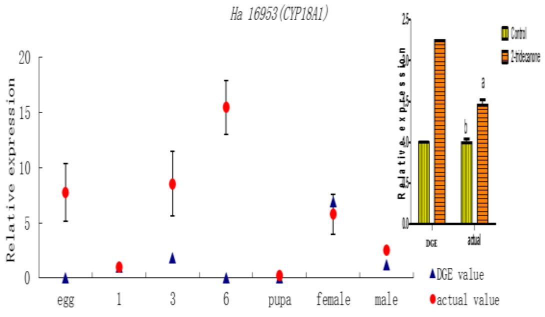

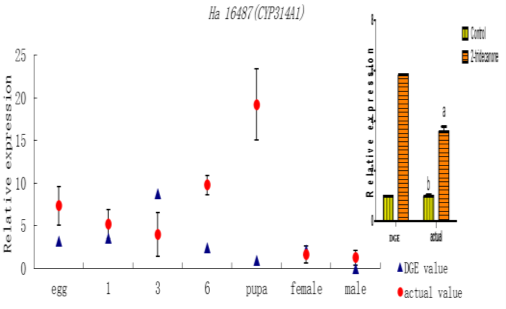

C: Invariable

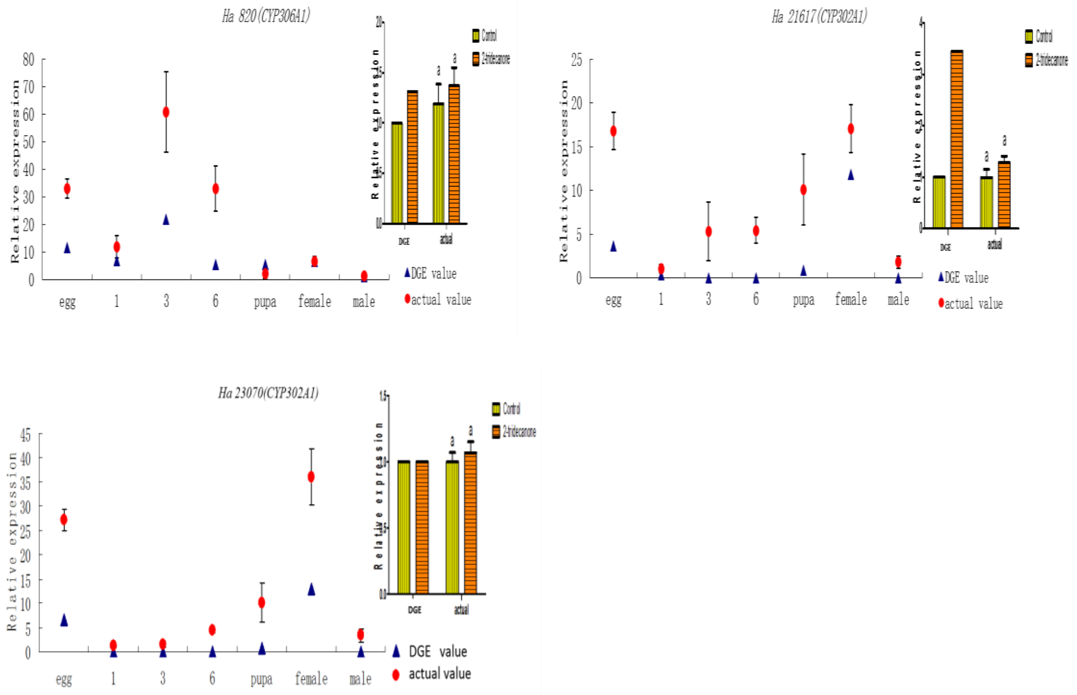

Fig. S5 Validation of hormone-regulated P450 genes expression by Real Time qPCR. (A) hormone-regulated P450 genes which were down-regulated by 2-TD. (B) hormone-regulated P450 genes which were up-regulated by 2-TD. (C) hormone-regulated P450 genes which were not significant inducible by 2-TD. 1st: first-instar larvae; 3rd: third-instar larvae; 6th: sixth-instar larvae; Control: sixth-instar larvae not treated with 2-tridecanone; 2-tridecanone: sixth-instar larvae treated with 10mg/g (w:w) 2-tridecanone for 24 hours. DGE values represent the expression of P450 unigene in DGE library; actual values represent the P450 unigene expression by qPCR. Statistical analyses were performed using GraphPad Prism 5.0 software. Statistical significance was determined using a Student's t-test, and p values less than 0.05 were considered as statistically significant. In each unigenes diagram, bars sharing the same letter in each group are not significantly different at $P > 0.05$.
