# Supplementary material for: The retardant effect of 2-Tridecanone, mediated by Cytochrome P450, on the Development of Cotton bollworm, Helicoverpa armigera
Source: BMC Genomics. 2016 Nov 22;17:954. doi: 10.1186/s12864-016-3277-y (PMC5118896; doi:10.1186/s12864-016-3277-y)
Supplement: Additional file 10: — Validation of gene expression by RT-PCR. (A) P450 unigenes which were regulated by 2-TD. (B) P450 unigenes which were not significantly induced by 2-TD. (C) P450 unigenes expressed at specific Developmental stages. (PDF 563 kb) [file 12864_2016_3277_MOESM10_ESM.pdf]

A:

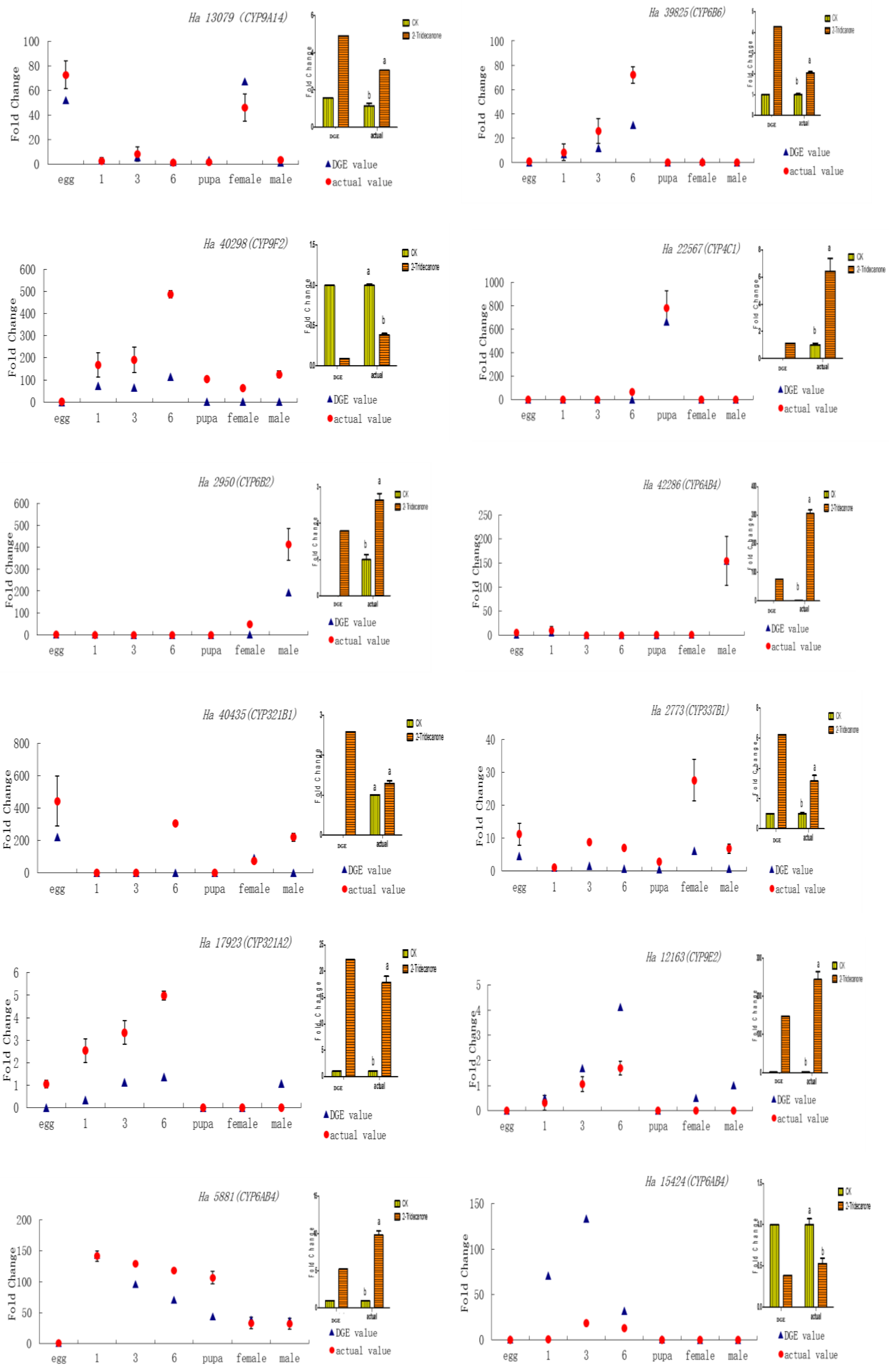
Ha 13079 (CYP9A14)
Ha 39825 (CYP6B6)
Ha 40298 (CYP9F2)
Ha 22567 (CYP4C1)
Ha 2950 (CYP6B2)
Ha 42286 (CYP6AB4)
Ha 40435 (CYP321B1)
Ha 2773 (CYP337B1)
Ha 17923 (CYP321A2)
Ha 12163 (CYP9E2)
Ha 5881 (CYP6AB4)
Ha 15424 (CYP6AB4)
Fold Change
egg
1
3
6
pupa
female
male
DGE value
actual value
CK
2-Tridecanone
DGE
actual
a
b

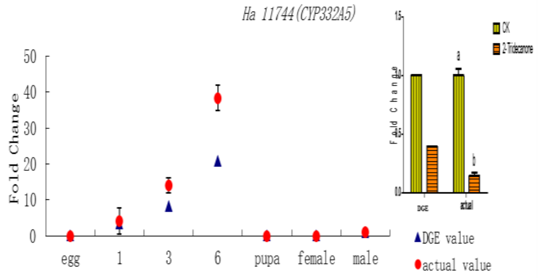
Ha 11744 (CYP332A5)
Fold Change
egg 1 3 6 pupa female male
DGE value
actual value
CK
2-Tridecanone
DGE actual

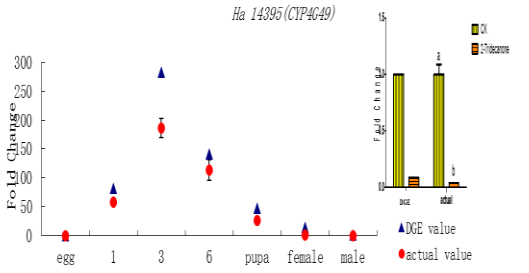
Ha 14395 (CYP4G49)
Fold Change
egg 1 3 6 pupa female male
DGE value
actual value
CK
2-Tridecanone
DGE actual

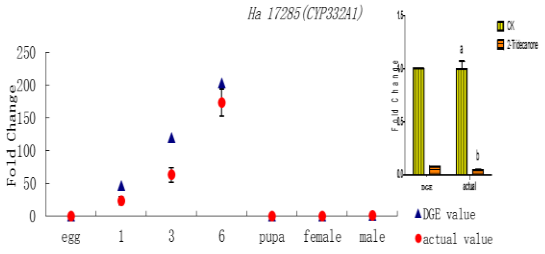
Ha 17285 (CYP332A1)
Fold Change
egg 1 3 6 pupa female male
DGE value
actual value
CK
2-Tridecanone
DGE actual

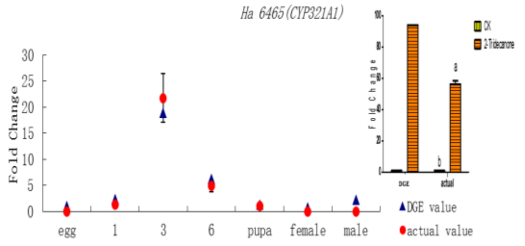
Ha 6465 (CYP321A1)
Fold Change
egg 1 3 6 pupa female male
DGE value
actual value
CK
2-Tridecanone
DGE actual

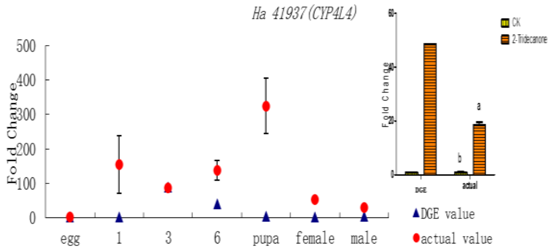
Ha 41937 (CYP4L4)
Fold Change
egg 1 3 6 pupa female male
DGE value
actual value
CK
2-Tridecanone
DGE actual

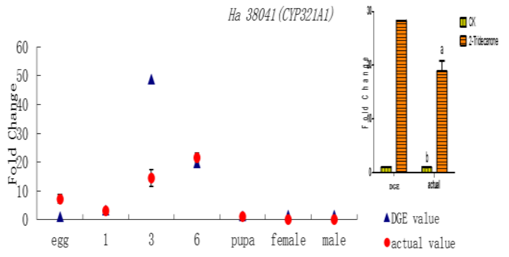
Ha 38041 (CYP321A1)
Fold Change
egg 1 3 6 pupa female male
DGE value
actual value
CK
2-Tridecanone
DGE actual

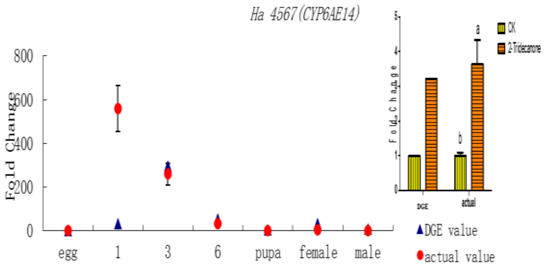
Ha 4567 (CYP6AE14)
Fold Change
egg 1 3 6 pupa female male
DGE value
actual value
CK
2-Tridecanone
DGE actual

B:

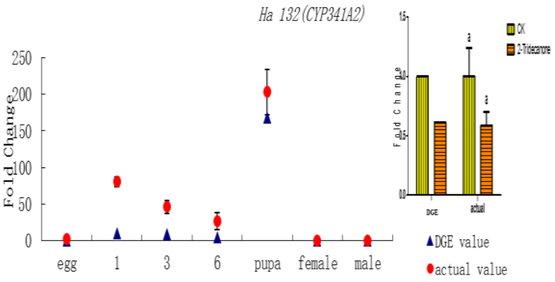
Ha 132 (CYP341A2)
Fold Change
egg 1 3 6 pupa female male
DGE value
actual value
CK
2-Tridecanone
DGE actual

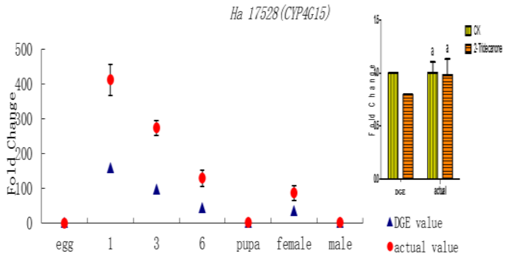
Ha 17528 (CYP4G15)
Fold Change
egg 1 3 6 pupa female male
DGE value
actual value
CK
2-Tridecanone
DGE actual

C:

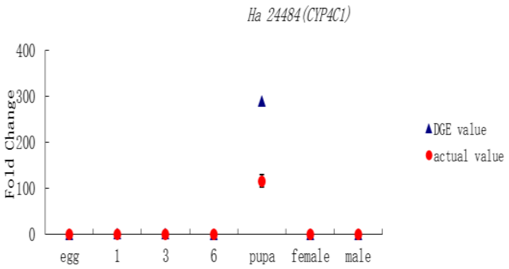

Fig. S6 Validation of gene expression by RT-PCR (A) P450 unigenes which were regulated by 2-tridecanone; (B) P450 unigenes which were not significantly induced by 2-tridecanone; (C) P450 unigenes expressed at specific Developmental stages. 1: 1st instar larvae; 3: 3rd instar larvae; 6: 6th instar larvae; CK: control groups. 2-tridecanone: sixth-instar larvae treated with 10mg/g (w:w) 2-tridecanone for 24 hours. DGE values represent the expression of P450 unigene in DGE library; actual values represent the P450 unigene expression by qPCR. Statistical analyses were performed using GraphPad Prism 5.0 software. Statistical significance was determined using a Student's t-test, and the p values less than 0.05 were considered as statistically significant. In each unigenes diagram, bars sharing the same letter for each group are not significantly different at $P > 0.05$.
