# Supplementary material for: The retardant effect of 2-Tridecanone, mediated by Cytochrome P450, on the Development of Cotton bollworm, Helicoverpa armigera
Source: BMC Genomics. 2016 Nov 22;17:954. doi: 10.1186/s12864-016-3277-y (PMC5118896; doi:10.1186/s12864-016-3277-y)
Supplement: Additional file 12: — Primers were used in this manuscript. (DOCX 18 kb) [file 12864_2016_3277_MOESM12_ESM.docx]

**Table Primers were used in this manuscript**

| **Gene** | **Primer name** | **Sequence（5’-3’）** | | **Efficiency** | **Amplicon size** |
| --- | --- | --- | --- | --- | --- |
| 11744 | 11744-F | 5’-AAGGAAGAAGGTGAGGGTGAACTC-3’ | | 99% | 303 bp |
|  | 11744-R | 5’-CAGATAAGACATGAAATAACACCAACA-3’ | |  |  |
| 14395 | 14395-F | 5’-CCGATCTCAGGCCTCATCTTCT-3’ | | 99% | 197 bp |
|  | 14395-R | 5’-CACATTGACAAGGCTTATGAATACA-3’ | |  |  |
| 5881 | 5881-F | 5’-TTACGAATGTTTCCTTCACTGGGCTT-3’ | | 101% | 218 bp |
|  | 5881-R | 5’-CGGCAAATAGACGAACTTATTATTGGAA-3’ | |  |  |
| 41937 | 41937-F | 5’-ATTTTGCATCCTCATCGTTACTATTGC-3’ | | 98% | 217 bp |
|  | 41937-R | 5’-GCCAGTATTTTGCAAGAATCAGAGATAT-3’ | |  |  |
| 39825 | 39825-F | 5’-GTCAATTTTTCCAACGGCGGTAA-3’ | | 99% | 213 bp |
|  | 39825-R | 5’-CTTGGGCACATATTACTTCATCTGTT-3’ | |  |  |
| 40298 | 40298-F | 5’-CATCATATTCCCTAATCTCCTGTGC-3’ | | 99% | 250 bp |
|  | 40298-R | 5’-GGTAAACTAACCCATGAAGATATAATGC-3’ | |  |  |
| 12163 | 12163-F | 5’-TGTAGATAAGGTGATAAATGAGGCGATG-3’ | | 96% | 199 bp |
|  | 12163-R | 5’-GAATCGCTCAGGATCAAACACTT-3’ | |  |  |
| 17923 | 17923-R | 5’-TAAGACATGCTGGACGTTCTTGG-3’ | | 98% | 177 bp |
|  | 17923-F | 5’-TACGGCAAGAACAAAGTGACAGGA-3’ | |  |  |
| 13079 | 13097-R | 5’-GGGAATGACTGGACCTGCATCA-3’ | | 99% | 211 bp |
|  | 13097-F | 5’-TCAAGATCCAAATGAAACAATGAAGAAT-3’ | |  |  |
| 21617 | 21617-F | 5’-GAAGAGGTAACGTGAAGAATTTTGAAG-3’ | | 100% | 299 bp |
|  | 21617-R | 5’-ATACACATGTGGTTTGTTTGTACGGTA-3’ | |  |  |
| 132 | 132-F | 5’-GATGACATCAATGAAGACCGTAGTAT-3’ | | 102% | 163 bp |
|  | 132-R | 5’-AGATCTCGCTTCTATTTGTATTTGGA-3’ | |  |  |
| 42286 | 42282-F | 5’-CCTGGCTCCACTGTGAATGC-3’ | | 100% | 120 bp |
|  | 42282-R | 5’-CAAATATGTGTTTTTGCCCTTTGG-3’ | |  |  |
| 24484 | 24484-F | 5’-CGAGGCGATAGAGAAAGGTGGACT-3’ | | 97% | 286 bp |
|  | 24484-R | 5’-GCTGAGCTGTTTCACCATGGT-3’ | |  |  |
| 15424 | 15424-F | 5’-GCGCTACACCACCGACTTCATC-3’ | | 101% | 195 bp |
|  | 15424-R | 5’-TCGAGGTCTTGTCCCAAAAACTTAAGTT-3’ | |  |  |
| 17528 | 17528-F | 5’-ACCCTCGTGACGTTGAGCTGAT-3’ | | 98% | 292 bp |
|  | 17528-R | 5’-GTTTCAAGCAAAATCTCGACGGT-3’ | |  |  |
| 2773 | 2773-F | 5’-CGACAATACCAACGCTCATTATAAAG-3’ | | 99% | 313 bp |
|  | 2773-R | 5’-GCTGTAGTGTATCTGGTCATAAGTTTG-3’ | |  |  |
| 2950 | 2950-F | 5’-AACCATCGCCATAGTAGTGATTTTACT-3’ | | 99% | 256 bp |
|  | 2950-R | 5’-CGCGATTTCGGGGTCTCTTATTATGA-3’ | |  |  |
| 38041 | 38041-F | 5’- TAACTACATCTCTAAGTAAACGTAACA -3’ | | 100% | 140 bp |
|  | 38041-R | 5’-TTGCTGTAGAACTTCACACCACGT-3’ | |  |  |
| 6465 | 6465-F | 5’-GACTGCTAATTAAACGAACTTACAAACA-3’ | | 100% | 129 bp |
|  | 6465-R | 5’-GCTAGTAGGTAATATAGCGATGTTTTGG-3’ | |  |  |
| 40435 | 40435-F | 5’-GTCAACCTCACTTGGACTTGTTC-3’ | | 97% | 125 bp |
|  | 40435-R | 5’-GCCAGGTACGCAAGAATTCAACTG-3’ | |  |  |
| 22567 | 22567-F | 5’-CTGAAAGTACAGGAATAATATCTATTGG-3’ | | 102% | 319 bp |
|  | 22567-R | 5’-GATGGGAGCTGTTTTTATTTACAATC-3’ | |  |  |
| 13311 | 13311-F | 5’-GATAAACTGTCAAACTGGTCTGCA-3’ | | 100% | 272 bp |
|  | 13311-R | 5’-GCCCTAACCTTTCTACCAACCTCA-3’ | |  |  |
| 8199 | 8199-F | 5’-CGGAAGAATATTGGCAAGAACCTGA-3’ | | 99% | 217 bp |
|  | 8199-R | 5’-CCTATGCATGTTCTTTTACCTATACTGA-3’ | |  |  |
| 13036 | 13036-F | 5’-CGGATGCTCGCGTGTGGTTTA-3’ | | 98% | 191 bp |
|  | 13036-R | 5’-GCATTTACTAATATCTACTCAATTCTCAC-3’ | |  |  |
| 820 | 820-F | 5’-CATCAGCCACATAGAACATTAGCTAA-3’ | | 99% | 244 bp |
|  | 820-R | 5’-GCCAGGTCGTAATTAACTTTCTTTGGT-3’ | |  |  |
| 21617 | 21617-F | 5’-TGTAATTCGATGATTCCGCTCTGTGA-3’ | | 98% | 228 bp |
|  | 21617-R | 5’-TCCCTACAACTGGATAACTCTTTGGT-3’ | |  |  |
| 23070 | 23070-F | 5’-AGTTTGAACACCCAGCTCTCCA-3’ | | 101% | 237 bp |
|  | 23070-R | 5’-GTATCATCTCGACTACAGCAGTTAA-3’ | |  |  |
| 40026 | 40026-F | 5’-AGGGCCGGAAATTAAGTACCTACCT-3’ | | 99% | 299 bp |
|  | 40026-R | 5’-CAGTTCCCCAGACCCGCTACCT-3’ | |  |  |
| 15409 | 15409-F | 5’-CGATTCGTTGATCTTGAACTGCATT-3’ | | 100% | 265 bp |
|  | 15409-R | 5’-TGAACTTAACTTCTTCATGACTCCA-3’ | |  |  |
| 16487 | 16487-F | 5’-AAGCTAACAAGGATACAACGATCGAT-3’ | | 100% | 231 bp |
|  | 16487-R | 5’-GACGTTACAGTTCTTTCGCGATATGAT-3’ | |  |  |
| 16953 | 16953-F | 5’-TATACAATCACTGAGTTCTCGGCAT-3’ | | 98% | 171 bp |
|  | 16953-R | 5’-GTCCCCACAATAGTTTAGAATTTGAAA-3’ | |  |  |
| 35643 | 35643-F | 5’-ATGGAGATGTTCATGTTCTTTGCGT-3’ | | 99% | 261 bp |
|  | 35643-R | 5’-ATCATTGAATACTGGTGGGTGTGTGTA-3’ | |  |  |
| 41217 | 41217-F | 5’-TCAGTTCCACCGTGACCACATA-3’ | | 96% | 226 bp |
|  | 41217-R | 5’-CAAAGAAGTCTCCATACCAGCAGAA-3’ | |  |  |
| 17285 | 17285-F | 5’-GTTGTATAACTTTACTTTTGACCTTTTA-3’ | | 98% | 161 bp |
|  | 17285-R | 5’-GATTATAAATTAAAAGCAAATTGTACCT-3’ | |  |  |
| 4567 | 4567-F | 5’-TTTGTATTGGTTTGAGATTCGCGA-3’ | | 99% | 177 bp |
|  | 4567-R | 5’-TCCTTCTCTTTCTATCATATTCAGTTTG-3’ | |  |  |
| *EF-α* | EF-1 | 5’-AGGAGTTGCGTCGTGGTTA-3’ | | 98% | 235 bp |
|  | EF-2 | 5’-GACTTGATGGACTTAGGGTTGT-3’ | |  |  |
| *CYP307A1* | qCYP307A1-F | 5'-ACAGCTATGAACATGTTTTCCCACT-3' | | 99% | 249 bp |
|  | qCYP307A1-R | 5'-CCAAATCTAACTCCCTCTGTTCTACA-3' | |  |  |
| **Application** | | **Primer name** | **Sequence（5’-3’）** | | |
| *CYP307A1* dsRNA synthesis | | dsRNAi-CYP307A1-1 | 5'-(T7)- ATATTCAGCCTGCAACTGGG-3'  5'-(T7)-GGCAAGAAATCCAAAGCGTA-3'  5'-(T7)-CAGTGCTTCAGCCGCTAC-3'  5'-(T7)-GTTCACCTTGATGCCGTTC-3' | | |
|  |  | dsRNAi-CYP307A1-2 |  |  |  |
| *GFP* dsRNA synthesis | | dsGFP-F |  |  |  |
|  |  | dsGFP-R |  |  |  |
