# Supplementary material for: The retardant effect of 2-Tridecanone, mediated by Cytochrome P450, on the Development of Cotton bollworm, Helicoverpa armigera
Source: BMC Genomics. 2016 Nov 22;17:954. doi: 10.1186/s12864-016-3277-y (PMC5118896; doi:10.1186/s12864-016-3277-y)
Supplement: Additional file 13: — Expression stability of the candidate reference genes under different conditions. (DOCX 13 kb) [file 12864_2016_3277_MOESM13_ESM.docx]

**Table Expression stability of the candidate reference genes under different conditions**

| **Condition** | **Reference Gene** | **Stability** | |
| --- | --- | --- | --- |
|  |  | **geNorm (M value)** | **Normfinder (M value)** |
| Development | *18S* | 0.96 | 0.83 |
|  | *GAPDH* | 1.29 | 1.27 |
|  | *EF-1a* | 0.89 | 0.81 |
|  | *β* actin | 0.88 | 0.79 |
| 2-Tridecanone | *18S* | 0.81 | 0.74 |
|  | *GAPDH* | 0.52 | 0.46 |
|  | *EF-1a* | 0.47 | 0.41 |
|  | *β* actin | 0.78 | 0.73 |
